# Central neurocytoma exhibits radial glial cell signatures with FGFR3 hypomethylation and overexpression

**DOI:** 10.1038/s12276-024-01204-3

**Published:** 2024-04-12

**Authors:** Yeajina Lee, Tamrin Chowdhury, Sojin Kim, Hyeon Jong Yu, Kyung-Min Kim, Ho Kang, Min-Sung Kim, Jin Wook Kim, Yong-Hwy Kim, So Young Ji, Kihwan Hwang, Jung Ho Han, Jinha Hwang, Seong-Keun Yoo, Kyu Sang Lee, Gheeyoung Choe, Jae-Kyung Won, Sung-Hye Park, Yong Kyu Lee, Joo Heon Shin, Chul-Kee Park, Chae-Yong Kim, Jong-Il Kim

**Affiliations:** 1 Department of Biomedical Sciences, Seoul National University Graduate School, Seoul, Republic of Korea; 2 Genomic Medicine Institute, Medical Research Center, Seoul National University, Seoul, Republic of Korea; 3 Department of Neurosurgery, Seoul National University College of Medicine, Seoul National University Hospital, Seoul, Republic of Korea; 4 Department of Neurosurgery, Seoul National University College of Medicine, Seoul National University Bundang Hospital, Seongnam-si, Gyeonggi-do Republic of Korea; 5 Department of Laboratory Medicine, Korea University Anam Hospital, Seoul, Republic of Korea; 6 The Marc and Jennifer Lipschultz Precision Immunology Institute, Icahn School of Medicine at Mount Sinai, New York, NY 10029 USA; 7 Department of Pathology, Seoul National University College of Medicine, Seoul National University Bundang Hospital, Seongnam-si, Gyeonggi-do Republic of Korea; 8 Department of Pathology, Seoul National University College of Medicine, Seoul National University Hospital, Seoul, Republic of Korea; 9 Lieber Institute for Brain Development, Johns Hopkins Medical Campus, Baltimore, MD 21205 USA; 10 Department of Neurology, Johns Hopkins School of Medicine, Baltimore, MD USA

**Keywords:** Cancer epigenetics, CNS cancer

## Abstract

We explored the genomic events underlying central neurocytoma (CN), a rare neoplasm of the central nervous system, via multiomics approaches, including whole-exome sequencing, bulk and single-nuclei RNA sequencing, and methylation sequencing. We identified *FGFR3* hypomethylation leading to *FGFR3* overexpression as a major event in the ontogeny of CN that affects crucial downstream events, such as aberrant PI3K-AKT activity and neuronal development pathways. Furthermore, we found similarities between CN and radial glial cells based on analyses of gene markers and CN tumor cells and postulate that CN tumorigenesis is due to dysregulation of radial glial cell differentiation into neurons. Our data demonstrate the potential role of FGFR3 as one of the leading drivers of tumorigenesis in CN.

## Genomic breakthrough: FGFR3 overexpression drives rare brain tumor

Central neurocytoma (CN, a rare brain tumor usually found in young adults) is not well understood due to its rarity. This study used advanced technology to analyze the genetic makeup of CN. The researchers found that CNs don't have any major repeated changes in their genes, gene combinations, or gene copies that could cause the tumor. Instead, they found that the increase in activity of the PI3K-AKT pathway (a pathway involved in cell growth) and changes in several pathways involved in nerve cell development are controlled by an increase in FGFR3 (a protein that helps cells grow and divide) in CNs. This research provides important information about the genetics of CN and could help guide future research and treatments.

This summary was initially drafted using artificial intelligence, then revised and fact-checked by the author.

Introduction

Central neurocytoma (CN) is a rare type of central nervous system (CNS) tumor that is located exclusively in the ventricles of the brain. Only ~0.1–0.5% of primary brain tumors are reported to be CN^1^. Due to its low incidence, no comprehensive genomic analysis of CN using modern genome analysis technology has been reported to date, although there have been some clinical and molecular investigations of its diagnosis, and treatment. Patients with CN typically present as young adults with symptoms of hydrocephalus caused by the unique intraventricular location of origin of the tumor, which hinders cerebrospinal fluid circulation^2^. Surgical resection remains the mainstay management strategy for CN, but good responses are also reported after radiotherapy or radiosurgery for small, recurrent, and residual tumors^3^. The prognosis of CN is usually excellent^4^.

CN was first described as a distinct neoplasm of the CNS in 1982 by Hassoun et al.^5^. It was established as a new entity of CNS tumor showing a unique histological pattern of both glial and neuronal differentiation of tumor cells^6–8^. Recent histological and cellular studies have investigated whether CN originate from adult neural progenitor cells situated in the subventricular zone (SVZ) around the lateral ventricles or the subgranular zone (SGZ) in the hippocampus based on the similarity of these cells to bipotential radial glial cells located in these regions^9^. However, sporadic studies based on genetic analysis have failed to discover any significant genomic events responsible for the development and progression of CN^10–12^. To date, investigations of the genetic elements underlying CN tumorigenesis have been inconclusive.

In the present study, we employed a multiomics approach to establish a genetic profile of intraventricular CN. Through whole-exome sequencing (WES) and bulk RNA sequencing (RNA-seq), we confirmed that CNs do not contain any major recurrent somatic mutations, gene fusions, or copy number alterations that are responsible for tumorigenesis. However, we found that upregulation of the PI3K-AKT pathway and alterations in several neuronal development pathways accompanied by enrichment of radial glial cell markers are orchestrated by *FGFR3* upregulation in CN. We confirmed the genetic characteristics of the tumor cells through single-nuclei RNA sequencing (snRNA-seq). Based on methylation profiling with methylation sequencing (methyl-seq), we conclude that hypomethylation at *FGFR3* CpG sites is the leading driver of *FGFR3* overexpression, which triggers downstream activation of the PI3K-AKT pathway that ultimately leads to CN development and progression.

## Materials and methods

### Sample collection

Fresh frozen tissue from a total of six cases of histologically confirmed CN and matched peripheral blood samples were used for next-generation sequencing analysis. Paired tumor and normal brain samples from three separate patients in the CN were collected for snRNA-seq (Online Resource, Supplementary Table 1). Normal brain tissue was obtained from the frontal lobe within the surgical corridor via the transcortical approach. All tumor and normal tissues were snap-frozen with liquid nitrogen during surgery, and white blood cells were isolated from blood. The samples were stored at −80 °C. Another 14 formalin-fixed paraffin-embedded tumor samples were collected for validation and employed to generate a tissue array. This study was approved by the institutional review board of Seoul National University Hospital (IRB No: H-1404-056-572). Informed consent was obtained from all patients.

### DNA and RNA extraction

DNA was extracted from the frozen tumor tissue and WBC samples with a Qiagen QIAamp DNA Mini Kit (Qiagen, Valencia, CA), and RNA was extracted from the frozen tumor tissue with an RNeasy Lipid Tissue Mini Kit (Qiagen, Valencia, CA) following the manufacturer’s protocol. The extracted DNA and RNA were subsequently sent to Macrogen, Korea, for WES, RNA-seq, and methyl-seq.

### Single-nucleus extraction

Nuclei were isolated from frozen paired tumor and normal brain tissue samples separately using the “Frankenstein” nucleus isolation protocol described previously^13^. Briefly, ∼40 mg of frozen tissue was homogenized in chilled Nuclei EZ Lysis Buffer (Sigma–Aldrich, St. Louis, MO), and the homogenate was filtered using a 35 μm cell strainer (Falcon, #352235). The solution was centrifuged at 500 × *g* for 5 min at 4 °C in a benchtop centrifuge. Nuclei were resuspended in EZ lysis buffer, centrifuged again, and equilibrated to nuclei wash/resuspension buffer (1× phosphate-buffered saline, 1% bovine serum albumin (BSA), 0.2 U/μL RNase Inhibitor). The nucleus washing procedure was repeated three times, after which staining with DAPI (10 μg/mL) or propidium iodide (PI) was performed. After isolation, the nuclei were sorted using a Bio-Rad S3e Flow Cytometer (Bio-Rad) in 10X Genomics reverse transcription reagents (without enzymes) to ensure that the nuclei were free of debris.

### Next-generation sequencing

After quality control of the samples, a sequencing library was prepared by random fragmentation of DNA or cDNA followed by 5′ and 3′ adapter ligation. Library preparation was performed using the SureSelectXT library prep kit for WES, the TruSeq standard mRNA LT sample prep kit for RNA-seq, and the SureSelect Methyl-Seq library prep kit for methyl-seq. WES, RNA-seq, and methyl-seq were performed using the Illumina platform at Macrogen, Korea. The generated BCL binary sequences were subsequently converted to raw FASTQ files utilizing the Illumina bcl2fastq package. For snRNA-seq, ~8500 single nuclei/sample were sorted directly into 25.1 μl of reverse transcription reagent from 10x Genomics Single Cell 3′ Reagents Kit V.3.1 (without enzyme). Libraries were prepared according to the manufacturer’s instructions (10x Genomics), and their quantity and quality were assessed using a Qubit 4 and Agilent 2100 Bioanalyzer. Samples with an RNA integrity number quality score >6 were selected for sequencing. The libraries were sequenced according to the manufacturer’s recommendation using a MiSeq system for quality checking and then with the NovaSeq system using 10X Genomics technologies at Psomagen, Inc. (USA).

### WES data processing

Paired-end reads were mapped to the GRCh37 reference genome. The genome was aligned with BWA-MEM (v.0.7.15)^14^. Reads were sorted and indexed with SAMtools (v.1.6), and duplicate reads were marked with Picard v.2.1.1(https://broadinstitute.github.io/picard/) to reduce the PCR duplication rate^15^. The reads were subjected to base recalibration and indel-realignment with GATK (v.3.8) for further analysis^16^.

### SNV and Indel calling

Somatic mutations and indels were detected by MuTect2, and germline mutations were detected by HaplotypeCaller; both tools were obtained from GATK (v.3.8). For somatic mutations, we used mutations that passed the MuTect2 filter. Both somatic and germline mutations were annotated with Cosmic 86, ExAC, and gnomAD via ANNOVAR^17–20^. Mutations located in exonic and splicing regions were selected. To verify significant mutations, the total read depth cutoff was set to ten, and the minor allele frequency filtering conditions were as follows: ExAC EAS < 0.01, gnomAD EAS < 0.01, and Korean < 0.01^21^.

### Copy number variant (CNV) calling

CNVs were identified with CNVkit and CoNIFER (v.0.2.2)^22,23^. After obtaining the RPKM with CoNIFER, the ZRPKM value was calculated, and the results were transformed into the tumor-to-normal log2 ratio to identify amplifications and deletions. Using CNVkit, we checked for CNVs at the gene and segment levels. A variant was defined as a deletion according to a log2 value ≤ − 0.4 and an amplification according to a log2 value > 0.3.

### Tumor mutation burden (TMB) calculation

The TMB was calculated as the number of mutations located in the coding region divided by the length of the coding region of RefSeq genes (~30 Mb)^24^.

### Normal brain dataset

Sequencing reads and meta-data from normal postmortem human brains were downloaded through Synapse.org from accession syn12299750^25,26^. These data originate from postmortem tissue homogenates of the dorsolateral prefrontal cortex gray matter approximating Brodmann area 46/9 in postnatal samples. RNA-seq libraries were constructed from high-quality RNA samples using an Illumina mRNA Sequencing Sample Prep Kit following the manufacturer’s protocol, and the final cDNA libraries were sequenced using the Illumina HiSeq 2000 platform with 100 bp paired-end reads after multiple levels of quality control.

### Bulk RNA-seq data processing

The transcriptome was aligned to the GRCh37 reference genome by using STAR (v.2.6.0.a)^27^. The expected counts and FPKM values were calculated with RSEM (v.1.3.1)^28^. The normalized FPKM was calculated as the log base 10-transformed FPKM, and each log (FPKM + 1) value was then subtracted from the mean log (FPKM + 1) value. The package DESeq2 was used to obtain differentially expressed genes (DEGs)^29^. DEGs were defined as those genes for which the absolute value of the log2 ratio was ≥2, the adjusted *P* value was less than 0.05 and the baseMean was ≥100. Gene Ontology (GO) enrichment analysis was performed with gProfiler (https://biit.cs.ut.ee/gprofiler/gost)^30^. Single sample gene set enrichment analysis was subsequently conducted with GenePattern (v 10.1.0)^31^.

### Gene fusion analysis

RNA fusion was detected with STAR-Fusion (v.1.4.0)^32^. Reads were aligned with STAR (v.2.6.0.a). We applied the following fusion criteria to confidently identify gene fusions: (1) one of the genes in the fusion had to be a protein-coding gene, (2) the spanning reads and junction reads were not “0”, and (3) duplicate fusions with the same read counts at the same position were excluded.

### snRNA-seq data processing

Reads were aligned to the GRCh37 genome reference by using CellRanger (v.5.0.1)^33^, which was obtained from 10x Genomics. The aligned reads were subsequently processed following the basic pipeline of Seurat (v.4.0.1)^34^ in RStudio (v.4.0.3). We filtered out cells for which the number of features was greater than 8500 or less than 200 and with mitochondrial counts greater than 3% for each sample. The six samples were integrated with Harmony (v.1.0)^35^. Counts were normalized with the “LogNormalize” method, after which the “FindNeighbors” function with 35 dims were used for UMAP in Seurat Cell; the cell types were classified using scHCL (v.0.1.1)^36^, further characterized using well-known neural lineage markers (Online Resource, Supplementary Table 2). We performed GO enrichment analysis with gProfiler to identify differences between tumor, radial glial/astrocyte-like, and neurons from normal brain and CN tumor samples. We visualized tumor-specific markers with nebulosa^37^. We conducted pseudotime analysis using three major cell types (radial glial/astrocyte-like, tumor, and neuron cells) with Monocle2^38–40^. PCA of radial glial/astrocyte-like cell cluster was performed using 20 components. For PC1, 26% of the variables were involved; for PC2, 16% were involved. Each sample was divided by PC1, and tumor and normal samples were divided by PC2.

### DNA methyl-seq data processing

The methylome was aligned to the GRCh37 reference genome by Bowtie2 (v.2.2.7)^41^. Methylated and unmethylated reads were detected by Bismark (v.0.20.0)^42^. The methylation ratio was calculated as the number of methylated reads on both strands (positive and negative) divided by the total number of reads. We extracted CpGs that intersected with the normal sample. The total number of intersected CpGs was 395,792. We obtained the average of each tumor-to-normal ratio for every CpG. A region with a difference between the normal and tumor average ratios greater than 0.4 was considered to be a DMR. We defined DMRs as hypomethylated if the average tumor ratio was less than 0.3 and as hypermethylated if the average tumor ratio was greater than 0.7. We considered a probe to be CN specific if the mean value for CN samples differed from the mean values for other CNS tumors by more than 0.4.

### Immunohistochemistry

Tissue cores (diameter, 2 mm) were obtained from CN (*n* = 14) and normal brain (*n* = 2) FFPE blocks and inserted into recipient tissue microarray (TMA) blocks (SuperBioChips, Seoul, Republic of Korea). The TMA blocks were cut into 3-µm-thick slices for immunohistochemistry (IHC). All antibodies used for IHC analysis in this study were purchased from Abcam, Inc. (Cambridge, MA). FGFR3 (ab10651, 1:250), PIK3R3 (ab235234, 1:10), and AKT1 (ab235958, 1:30) were used as PI3K-AKT pathway markers. PAX6 (ab195045, 1:50), SOX2 (ab92494, 1:20), and FABP7 (ab110099, 1:5) were used for radial glial cells. SOX10 (ab180862, 1:200) was used for neuroepithelial cells. EOMES (ab23345, 1:20) and ASCL1 (ab213151, 1:50) were used for intermediate progenitor cells. NEUROD1 (ab213725, 1:500) was used for immature neurons. SYP (ab32127, 1:2000) was used for mature neurons. Appropriate positive controls were used according to the manufacturer’s instructions, and primary antibodies were omitted as negative controls. Two pathologists (JKW, SHP) reviewed the IHC slides and scored each sample on a negative to 4+ positive basis. Independent image quantification was carried out for each image with ImageJ Fiji software following methods described previously^43^.

### Statistical methods

The Wilcoxon test was used to compare the means of normal and tumor samples, and the *P* value is labeled with an asterisk. Error bars were calculated as the mean ± standard error; minimum error bars refer to the mean − standard error and maximum error bars are the mean + standard error.

### Telomere repeat amplification protocol (TRAP) assay with ELISA

The enzymatic activity of telomerase was measured using TeloTAGGG Telomerase PCR ELISA PLUS Kit (Roche) according to the manufacturer’s protocol. Tumor tissues were homogenized in ice-cold lysis buffer using an automill (Tokken). Briefly, after BCA protein quantification, 10 μg of protein was incubated in a 50 μl reaction mixture at 25 °C for 30 min to allow the telomerase to add telomeric repeats to the end of the biotin-labeled primer. PCR was conducted for 33 cycles of 94 °C for 30 s, 50 °C for 30 s, and 72 °C for 90 s, followed by an additional extension time of 10 min at 72 °C and holding at 4 °C. Telomerase activity was measured at 450 nm and at the reference wavelength of 690 nm. The relative telomerase activity of each sample was calculated according to the instructions of TeloTAGGG Telomerase PCR-ELISA PLUS Kit.

### C-circle assay

Detection of C-circles was performed as previously described (PMID: 27595911). Briefly, 30 ng of DNA was combined with 10 μl of 2× Φ29 Buffer, 7.5U of Φ29 DNA polymerase (NEB), 0.2 mg/ml BSA, 0.1% (v/v) Tween 20, and 1 mM each of the dATP, dGTP, and dTTP, and the mixture was incubated at 30 °C for 4 h and 8 h followed by 20 min at 70 °C. Amplification products were deposited on a Hybond N+ nylon membrane (Bio-Rad) and developed using TeloTAGGG Telomere Length Assay Kit (Roche). Chemiluminescent signals were visualized with a ChemiDoc XRS system (Bio-Rad).

### Telomere length fragmentation assay

Telomere length was determined by Southern blotting using a TeloTAGGG Telomere Length Assay Kit (Roche) according to the manufacturer’s protocol. Briefly, 1 µg of DNA was digested with RsaI and HinfI for O/N at 37 °C. The products were subjected to electrophoresis on a 0.8% agarose gel at 50 V for 4 h and then transferred to a nylon membrane by Southern blotting. The membrane was blocked and hybridized to a digoxigenin (DIG)-labeled probe specific for telomeric repeats for O/N. The washed blot was incubated with anti-DIG-alkaline phosphatase (1:1000 dilution) for 30 min and developed using substrate from a TeloTAGGG Telomere Length Assay Kit (Roche). Then, chemiluminescent signal images were captured with a ChemiDoc XRS system (Bio-Rad). TeloTool version 1.3 was used for image analysis and telomere length calculation.

### Visualization methods and statistics

The selected 10K probes were the most variable probes for classifying CNS tumors (DKFZ). Methylation values corresponding to 10K probes were used to generate the t-SNE plot with Rtsne^44^. A circus plot was generated with Circa (http://omgenomics.com/circa/). Illustrated figures were created with the help of BioRender.com. All statistical analyses were carried out using R. The Wilcoxon test was used to compare expression levels between normal and tumor tissue samples, and a linear model was used to determine the correlation between expression and methylation.

## Results

### Absence of major genetic alterations identified in CN

**Fig. 1:**
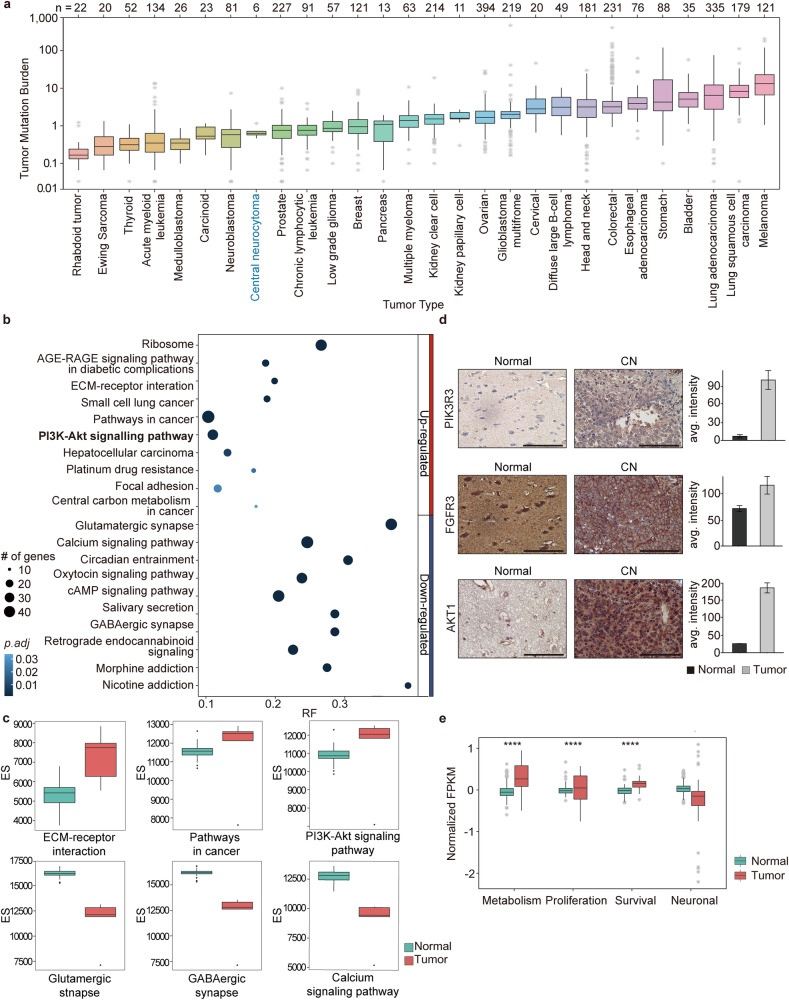
PI3K-AKT pathway activation in CN. **a** Comparison of the tumor mutation burden ratio of CN with other cancers revealed a low score relative to that of other CNS tumors, such as GBM and LGG. **b** Pathway analysis of significantly differentially expressed genes in CN compared to that in normal brain samples revealed the PI3K-AKT1 pathway as one of the top upregulated pathways in CN. **c** Enrichment analysis of significantly overexpressed genes in CN compared to that in normal brain samples. **d** IHC validation of overexpressed PI3K-AKT pathway marker genes along with *FGFR3* in CN (scale bar 100 µM). **e** Functional classification of the PI3K-AKT1 pathway downstream of GSK3-related genes showed that metabolism-, proliferation-, and survival-related genes were upregulated and that neuronal function-related genes were downregulated in CN. (Asterisks represent the following *P* values: *<0.05; **<0.01; ***<0.001; ****<0.0001).

We performed multiplatform genome profiling of six CN patient samples to determine the landscape of genomic alterations (Online Resource, Supplementary Fig. 1). Genome-wide profiling revealed no recurrently mutated genes or gene fusions (Online Resource, Supplementary Tables 3, 4). Copy number variation calling did not reveal any significant area of recurrent gains or losses in any chromosome (Online Resource, Supplementary Fig. 2). Commonly observed genomic alterations previously reported in various types of CNS tumors, such as *IDH1, IDH2, TP53, NF1, SMARCB1, FUBP1,* and *ATRX* mutations*, PTEN* deletion, *EGFR* amplification, and 1p/19q deletion^45^, were also checked and found to be absent in CN. Previous studies of CN involving karyotyping and target sequencing were also unable to establish any key genomic events as drivers of CN tumorigenesis^10,46–50^. In light of these data, we posited that specific genomic alterations might not initiate CN tumorigenesis. We also calculated the tumor mutation burden (TMB) for CN and compared it with that of 3083 previously published tumor datasets from 27 tumor types (Fig. 1a)^24^. The TMB of CN was much lower than that of glioblastoma (GBM) but closer to, though still lower than that of lower-grade glioma (LGG); the TMB of CN was 0.63, whereas that of GBM and LGG was 2.03 and 0.87, respectively. This finding was in line with the benign clinical characteristic of CN, as the TMB is considered a marker of the malignant progression of cancer^51^.

### Aberrant activity of the PI3K-AKT pathway in CN

Gene expression profiling of CNs and analysis of DEGs compared with age-matched normal brain data revealed the PI3K-AKT pathway to be the most significantly aberrant pathway among oncogenic pathways in CN (Fig. 1b, c and Online Resource, Supplementary Tables 5, 6). Upon closer inspection of the gene list obtained through pathway analysis, we observed that the *FGFR3*, *PIK3R3*, and *AKT1* genes were overexpressed in CN and responsible for the aberrant activity of the PI3K-AKT pathway. Additionally, we compared the RNA expression patterns of CN with those revealed by previously published microarray data^11,12^ and found that most of the upregulated genes identified in our data matched previously reported genes, including *FGFR3* and *PIK3R3* (Online Resource, Supplementary Table 7). Overexpression of the *FGFR3*, *PIK3R3*, and *AKT1* genes in CN was confirmed at the protein level using immunohistochemistry (IHC) in an independent validation set of 14 CNs (Fig. 1d and Online Resource, Supplementary Fig. 3). However, no significant changes in expression of genes downstream of *AKT1* (*FOXO* and *GSK3*) were detected in CN, except for the genes downstream of *GSK3* (Fig. 1e and Online Resource, Supplementary Fig. 4). Functional annotation of the downstream genes related to *GSK3* classified them into metabolism, proliferation, survival, and neuronal function categories^52^. In CN, metabolism-, proliferation-, and survival-related genes were mostly upregulated, while neuronal function-related genes were downregulated (Fig. 1e). Additionally, we checked gene sets related to neurodevelopment (neuronal differentiation, neural projection guidance, neural precursor proliferation, etc.) and found that most of the genes were downregulated in CN, except for genes involved in the neural precursor proliferation pathway (Online Resource, Supplementary Fig. 5).

### Enrichment of radial glial cell markers in CN

**Fig. 2:**
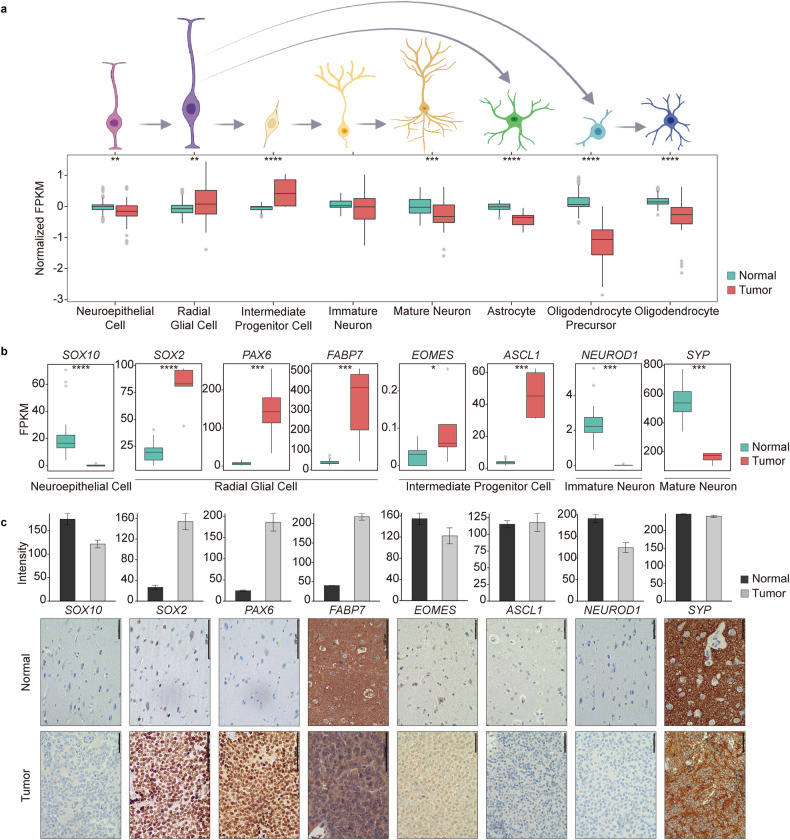
CN shows a similar gene expression pattern to that of radial glial cells in the bulk RNA sequencing data. **a** Upper schematic figure showing various stages of neural precursor cells in the normal brain and their progression. The arrow shows the expected paths of the cells during normal neural differentiation. Lower bar charts show the combined gene expression patterns of these different neural developmental cell marker genes in CN and normal brain samples. Significant overexpression of genes related to radial glial and intermediate progenitor cells was detected in CN (asterisks represent the following *P* values, *<0.05; **<0.01; ***<0.001; ****<0.0001). **b** Individual marker gene expression patterns showing significant overexpression of all marker genes representing radial glial cells and intermediate progenitor cells in CN compared to the normal brain (asterisks represent the following *P* values, *<0.05; **<0.01; ***<0.001; ****<0.0001). **c** IHC validation of radial glial and intermediate progenitor cell marker genes showing that only radial glial marker genes are upregulated in CN, confirming that radial glial cells are the cell type of origin (scale bar 100 µM).

Considering that a set of neuronal function-related genes are downregulated and that neural precursor proliferation-related genes are upregulated in CN, we investigated deeper into expression of different neural progenitor cells in CN. The neural progenitor lineage can differentiate into either neural or glial cells depending on environmental cues. Radial glial cells can differentiate into mature neurons or glial cells, such as astrocytes or oligodendrocytes (Fig. 2a, Online Resource, Supplementary Table 2). To identify the cell type of origin of CN, we explored well-known gene markers for each of these neural progenitor cell types previously reported in multiple studies (Fig. 2b, Online Resource, Supplementary Table 8) and corroborated the findings at the protein level by IHC with a 14 CN sample tissue array validation set (Fig. 2c and Online Resource, Supplementary Fig. 3). We confirmed that at the RNA level, CN showed decreased expression of genes related to neuroepithelial cells, mature neurons, immature neurons, astrocytes, and oligodendrocytes but increased expression of radial glial cells and intermediate progenitor cell-related genes (Fig. 2a). However, when we examined the gene expression patterns of each cell type, we observed that CN exhibited more prominent enrichment of the radial glial cell signature than the intermediate progenitor cell signature. *SOX2, PAX6,* and *FABP7*, which are used as radial glial cell markers, exhibited significant analogous expression patterns at both the RNA and protein levels, whereas *EOMES* and *ASCL1*, which are used as markers of intermediate progenitor cells, exhibited different patterns (Fig. 2b, c). *EOMES* was minimally overexpressed in CN samples compared to normal brains at the RNA level but not at the protein level, which was not surprising considering the minimal difference in RNA expression (Fig. 2b, c). *ASCL1* was considerably overexpressed in CN at the RNA level but not at the protein level. *SOX10*, *NEUROD1,* and *SYP* were expressed at lower levels in CN than in the normal brain according to both RNA-seq and IHC validation. In view of these results, we determined that the origin of CN is most likely to be radial glial cells rather than intermediate progenitor cells.

### Characterization of tumor cells in CN

**Fig. 3:**
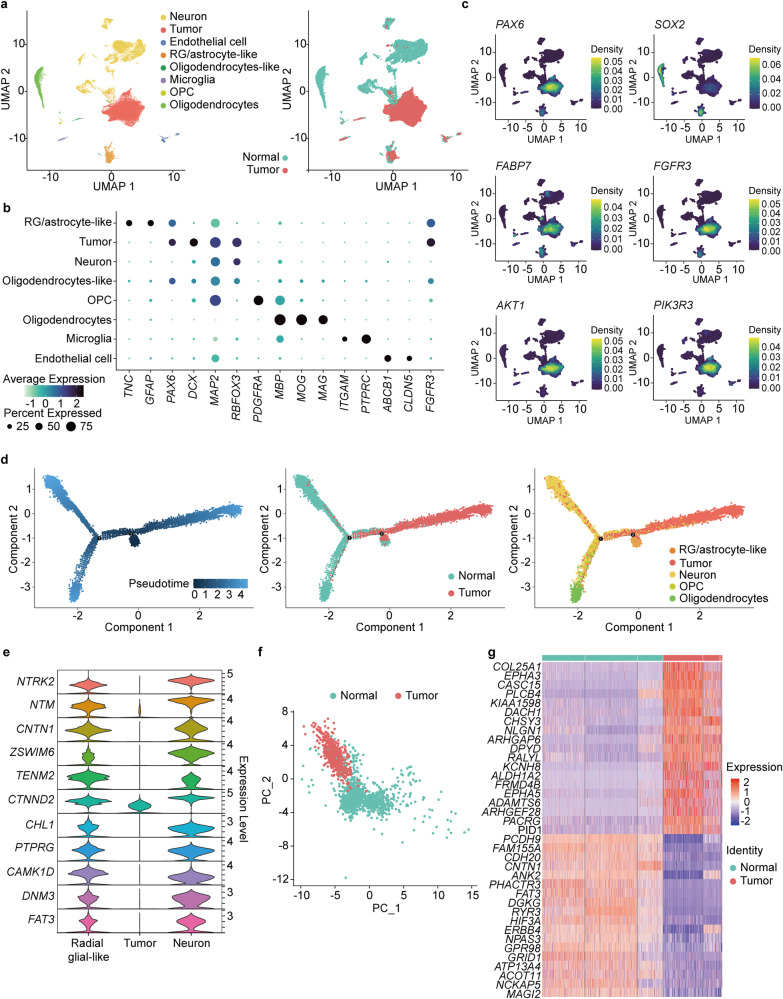
Radial glial-like cells deviate from their normal developmental course and give rise to CN. **a** snRNA-seq analysis of paired CN tumor and normal brain samples. **b** Cell-specific gene markers for each identified cell type are shown. *FGFR3* can be detected in both tumor and radial glial cells. **c** Confirmation of overexpression of *FGFR3*, the PI3K-AKT1 pathway, and radial glial cell markers in tumor cell clusters consistent with the bulk RNA-seq analysis. **d** Pseudotime analysis of tumor cells, neurons, oligodendrocytes, and radial glial/astrocyte-like cells showing tumor cells positioned between neurons and radial glial/astrocyte-like cells. **e** Genes expressed in both neurons and radial glial/astrocyte-like cells but not in tumor cells. **f** PCA plot showing that despite being identified as the same cell type, radial glial/astrocyte-like cells from tumor and normal samples show some differences. **g** Heatmap of differentially expressed genes in normal and tumor sample radial glial/astrocyte-like cells.

To establish the cell population that constitutes CN, we conducted snRNA-seq of three CN tissue samples with paired normal brain tissue samples obtained during resection via a transcortical approach. A total of 32,945 cells, which consisted of 17,122 normal cells and 15,823 tumor cells, were used in the analysis. We classified clusters of cells based on previously published single-cell references from brain tissues and according to well-known markers (Online Resource, Supplementary Table 2)^36,53,54^. Radial glial cells and astrocytes share common markers, such as GFAP and SLC1A3. Therefore, we isolated cluster 5 and examined the expression levels of each marker gene in detail (Supplementary Fig. 6). Both markers for radial glial cells and astrocytes were expressed in normal samples, whereas radial glial cell markers were predominantly expressed in CN. As expected, the major cell clusters in the normal brain samples were classified as radial glial/astrocyte-like (*n* = 1138), oligodendrocyte (*n* = 3334), or neuron (*n* = 11,484) (Fig. 3a), and the minor clusters included microglia, endothelial cells, OPCs, oligodendrocyte-like cells and some tumor cells (Online Resource, Supplementary Table 9). In contrast, the major clusters of tumor samples included tumor cells (*n* = 14,663) and radial glial/astrocyte-like cells (*n* = 552), and the minor populations included neurons, microglia, OPCs, oligodendrocyte-like cells, and endothelial cells (Fig. 3a, Online Resource, Supplementary Table 9). Oligodendrocyte-like cell clusters were split into two groups: one consisted of normal sample cells (*n* = 66), and the other was enriched with tumor sample cells (*n* = 130). We did not find any oligodendrocyte cells among the tumor samples.

We identified highly expressed genes in each cluster, and *FGFR3* was found in both the radial glial/astrocyte-like and tumor cell clusters but not in the neuron cell cluster (Fig. 3b). We further investigated the tumor cell population to determine whether intratumoral heterogeneity existed in the CN samples and found that there were six clusters (clusters 0, 2, 7, 8, 10, and 14) annotated as tumor cells in the CN samples, among which two clusters (clusters 0 and 2) accounted for the majority of the tumor cell population. We confirmed the presence of clusters 0, 2, and 10 in all three tumor samples individually (Online Resource, Supplementary Fig. 7). Tumor cell Clusters 7, 8, and 14 were confirmed to be sample specific, as they were not present equally in all three CN tumor samples (Online Resource, Supplementary Fig. 7); moreover, we could not identify enriched genes in these clusters. These minor tumor cell clusters can be considered to either represent sample bias or be a sign of intertumoral heterogeneity. We also checked expression of the genes that we previously confirmed via bulk RNA-seq to be CN specific. Expression of radial glial cell signature genes (*PAX6, SOX2, FABP7*) and *FGFR3* was confirmed in both the radial glial/astrocyte-like and tumor cell clusters, whereas *PIK3R3* and *AKT1* were only expressed in the tumor cell cluster (Fig. 3c). *SOX2* was expressed at lower levels in the single-cell tumor clusters than in the bulk RNA-seq data. According to snRNA-seq data, *SOX2* is overexpressed in tumors and in radial glial/astrocyte-like and oligodendrocyte cell clusters. However, compared to that in tumor cell clusters, overexpression of *SOX2* in radial glial/astrocyte-like and oligodendrocyte cell clusters was more notable. These results further corroborate our identification of radial glial cells as the likely origin of CN and PI3K-AKT pathway activation according to bulk RNA-seq results.

### Radial glial cells in limbo arbitrarily develop into CN

To investigate the tumorigenesis of CN, we investigated DEGs and corresponding pathways between radial glial, neurons, and tumor cells in single-cell clusters. Consistent with the bulk RNA-seq findings, the PI3K-AKT pathway was found to be enriched in the snRNA-seq tumor cell cluster (Online Resource, Supplementary Fig. 8). We also observed that the neuronal differentiation pathway was enriched in both tumor and neuron cell clusters, but intriguingly, different sets of genes were responsible for enrichment of this pathway in tumor and neuron cells (Online Resource, Supplementary Table 10). These findings compelled us to consider that CN tumor cells might result from deviation of radial glia from the normal neuron differentiation course. Therefore, to explore this possibility, we next performed trajectory analysis with the three major cell populations of interest (tumor, radial glial, and neuron cells) to determine whether there is any difference in these populations according to their cell differentiation status (Fig. 3d and Online Resource, Supplementary Table 11). Indeed, we observed that the tumor cells were positioned between the radial glial and neuron populations, indicating that CN tumor cells were more differentiated than radial glial cells but less differentiated than neurons (Fig. 3d, Online Resource, Supplementary Fig. 9). We speculated about the possible pathways that might play an active role in driving radial glial cells to deviate from their natural differentiation course to become CN tumor cells and analyzed GO enrichment of genes rarely expressed in tumor clusters but differentially expressed in radial glial cell and neuron clusters (Online Resource, Supplementary Table 10). As a result, we observed that neuron-related genes (*CNTN1, FAT3, PTPRG, NTRK2*, etc.) annotated to categories such as nervous system development, neuron differentiation, generation of neurons, and neurogenesis were downregulated in tumor cluster cells (Fig. 3e, Online Resource, Supplementary Table 10). Additionally, we noted that tumor and normal sample cells in the radial glial/astrocyte-like cluster grouped together according to the UMAP plot for all cell types; however, they clustered separately according to pseudotime analysis. This observation prompted us to investigate differences between tumor sample radial glial cells and normal sample radial glial cells. Hence, to identify genes differentially expressed between tumor and normal samples in the radial glial/astrocyte-like cluster, we conducted principal component analysis with only the radial glial cell type, and each cell was divided by PC2 (Fig. 3f). It was thus possible to identify genes (*CNTN1, FAT3, EPHA3, EPHA5, NLGN1*, etc.) that contribute to PC2 and are differentially expressed in radial glial cells between normal and tumor samples (Fig. 3g). According to these findings, we conclude that CN mainly originates from radial glial/astrocyte-like cells that deviate from the natural course of radial glial-to-neuronal differentiation and develop into tumor cells, which involves dysregulation of several significant pathways essential for normal radial glial-to-neuronal differentiation.

### Epigenetic hypomethylation of FGFR3 in CN

**Fig. 4:**
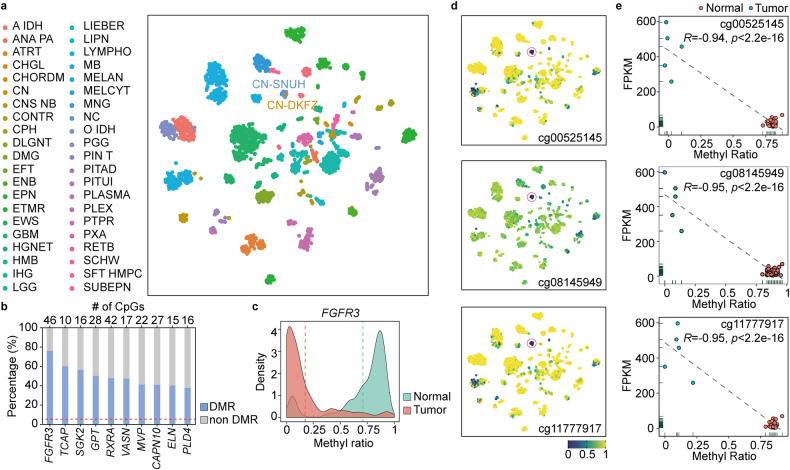
FGFR3 hypomethylation in CN. **a** t-SNE clustering based on methylation data showing that the CN samples from this study clustered exclusively with the public CNS data from the CN cluster, confirming accurate classification of the samples as CN. CN-SNUH represents the samples from the present study (*n* = 6), and CN-DFKZ represents the samples from comparison of public methylation data (*n* = 21). **b** The *FGFR3* gene had the highest percentage among DMR/non-DMR ratios. **c** *FGFR3* was hypomethylated compared to normal brain samples according to methylation expression data. **d** *FGFR3*-specific CpG probes (*n* = 3) showed significant hypomethylation compared to that in other CNS tumors and normal brain samples. The CN samples indicated by red circles contain 6 samples from the present study and 21 samples obtained from the public DFKZ CNS methylation database. **e** Correlation plot of methylation ratios with FPKM values showing a significant negative correlation for all three FGFR3-specific probes.

We also explored the epigenetic profile of CNs through methyl-seq. To confirm the DNA methylation-based classification of CNs, we used 8535 common probes from our targeted bisulfite sequencing data that matched with the 10,000 most variable core probes from the 450K methyl array data; these probes were selected to classify CNS tumors based on the DFKZ classifier^55^. All patients included in this study clustered perfectly with those in the DKFZ CN group according to t-SNE analysis (Fig. 4a). To verify whether the changes in methylation patterns affect gene expression, we investigated the DNA methylation levels of CpGs within the DEGs by applying the selection criteria: genes with more than 10 CpGs, and more than half are differentially methylated regions (DMRs). Among the 167 DEGs with implemented DMR criteria, the top ten genes were selected according to the highest DMR ratios, and *FGFR3* was found to be the most differentially methylated gene (median DMR 0.7, mean DMR 0.54) (Fig. 4b, c). Among the 46 probes within *FGFR3*, 42 CpGs were included in the DKFZ most variable 10K list, and as many as 76% of the CpGs in *FGFR3* were hypomethylated in CN (Fig. 4b). Then, we checked the methylation level of the *FGFR3* gene within the DKFZ CNS tumors to determine whether *FGFR3* hypomethylation is a CN specific event. The three most variable probes (cg00525145, cg08145949, and cg11777917) showing differences in methylation ratios greater than 0.8 relative to the normal level were selected (Fig. 4d and Online Resource, Supplementary Table 12). In addition to the 3 CpGs, we observed probes among the other DEGs that exhibited CN-specific methylation or unmethylation relative to the data from other CNS tumors and normal brains (Online Resource, Supplementary Fig. 10). Furthermore, methylation of the three most variable probes correlated strongly negatively with *FGFR3* expression (Fig. 4e). This correlation of epigenetic and transcriptomic data for *FGFR3* specifically indicated the potential of *FGFR3* as a major driver of CN oncogenesis.

### Telomerase maintenance mechanism in CN

None of the CN patients examined in the present study presented any mutations in the *TERT* promoter or *ATRX* region, which are frequently observed in glioma. To evaluate the active telomere maintenance mechanism (TMM) in CN, we first checked *TERT* expression and found that none of the CN samples expressed *TERT* (Online Resource, Supplementary Fig. 11a). Telomerase activity was measured in CN tissues by PCR-ELISA, and we confirmed that all CN tissues exhibited very low telomerase activity (Online Resource, Supplementary Fig. 11b). We also checked the CN samples with a C-circle assay and found that all of the CN samples presented negative results, implying that no alternative lengthening of telomeres phenomenon occurs in CN (Online Resource, Supplementary Fig. 11c). The mean telomere length of CN measured by southern blotting ranged from 9 to 13 kb on average (Online Resource, Supplementary Fig. 11d), and the range of telomere length detection was between 20 and 30 kb on average. The telomere length in the tumors showed little variation according to patient age and exhibited a homogenous intertumoral pattern (Online Resource, Supplementary Table 13). According to previous studies, the telomere length in normal brain tissue has been reported to be more than 10 kb, in contrast to what has been found in other types of astroglial brain tumors (<10 kb)^56^. Taken together, these results indicate that the TMM does not actively operate in CN, suggesting the benign nature of its biological behavior.

## Discussion

CN is a unique type of CNS tumor in terms of its typical intraventricular location. CN was first included in the WHO Classification of CNS Tumors as a separate entity in 1993^8^ and still holds its position in the present 2021 WHO Classification under the category of glioneuronal and neuronal tumors^57,58^. The biological behavior of CN is reported to be mostly benign, but some aggressive types also exist^49,59^. The origin of CN was first proposed in 1991 by von Deimling et al., who indicated that it originates from the bipotential progenitor cells of the SVZ in the adult brain^9^. Later studies revealed the dual characteristics of both neuronal and glial cells based on in vitro primary cultures of CN tumor cells^6,60^. However, due to the rarity of these tumors, research related to CN is limited, especially in regard to comprehensive genomic studies using the most recent technology. To date, only six studies involving CN have included sequencing results (based on microarray, karyotyping, and target sequencing)^10,46–50^. In this study, to address this knowledge gap, we performed genomic profiling of CN using next-generation sequencing technologies such as WES, bulk and snRNA-seq, and methyl-seq to determine the key elements underlying CN development.

We confirmed that our CN samples did not harbor any recurrent somatic mutations, copy number variant regions, or gene fusions driving tumorigenesis. This result was consistent with the failure to find a genomic event serving as a universal driver of CN in previous studies^10–12^. We then focused on our transcriptomic data and GO pathway analysis of DEGs based on both bulk RNA-seq and snRNA-seq data, which revealed the altered PI3K-AKT pathway as a consistent candidate for CN tumorigenesis. The relevant genes included *FGFR3*, *PIK3R3*, and *AKT1*, all of which play major roles in activation of the PI3K-AKT pathway and oncogenesis^61,62^. Among the downstream genes of the PI3K-AKT pathway, those related to *GSK3*, notably including neuronal function-related genes, were significantly downregulated, which was consistent with the findings of previously published studies^63,64^. We also detected aberrant activity in neural developmental pathways. Pathway analysis revealed upregulation of the neural precursor cell proliferation pathway. After checking expression of marker genes related to neuronal cell stages, we confirmed the radial glial cell-related marker genes *PAX6*, *SOX10,* and *FABP7* to be significantly upregulated in CN tissue samples. Studies based on CN tumor spheres showed phenotypic similarity of CN tumor cells to radial glial cells and neural progenitor cells, suggesting that CN cells might originate from radial glial cells located in the adult SVZ and SGZ^11,65^.

We also noted that pathways related to neurodevelopment, such as neuronal development, differentiation, generation, and neurogenesis, were downregulated in CN. It has been previously established that neurogenesis occurs in the SVZ and SGZ of the brain^66^. During this process, neuroepithelial cells differentiate from radial glial cells. These cells can be differentiated into both neuronal cells and glial cells depending upon environmental cues. Radial glial cells in the SVZ follow the rostral migratory system and migrate first toward the olfactory bulb and ultimately to the frontal cortex, where they give rise to either neurons or glial cells^67^. In addition, radial glial cells in the SGZ follow a short path from the dentate gyrus toward the hippocampus^67^. Neurodevelopmental pathways play a crucial role in these migration processes to guide cells toward the correct path^68^. Downregulation of these pathways might cause radial glial cells to deviate from their original course and migrate more centrally into ventricles. This hypothesis was supported by our snRNA-seq analysis, in which we clearly observed that genes related to neuronal migration and axon guidance (*CNTN1, PTPRG, NTRK2, FAT3*, etc.) were more upregulated in neuron cell clusters than in tumor cell clusters. The *CNTN1* gene has been previously reported to play an important role in neuronal migration, and PTPRG-CNTN signaling has been indicated to be a critical mechanism for normal neuronal development^69,70^. Interestingly, a single alteration in the *FAT3* gene was shown to be sufficient to cause fundamental changes that drive CNS evolution in a previous study^71^. Furthermore, our analysis identified several genes differentially expressed in radial glial cells between normal and tumor samples, among which Eph receptor-related genes, such as *EPHA3* and *EPHA5*, were expressed in the radial glial cells of tumor samples but not in the radial glial cells of normal brain samples. This is a compelling finding because the role of the Eph receptor family in tumorigenesis has been well established in previous research, and crosstalk between the Eph receptor and PI3K-AKT signaling has also been reported^72^. Whether overexpression of Eph receptor-related genes plays any role in activation of the PI3K-AKT pathway and tumorigenesis in CN, in addition to the role of *FGFR3*, is another exciting question that should be explored in the future.

The key finding of the present study is CN-specific hypomethylation of 3 CpG sites in *FGFR3*. This epigenetic change might be the main cause underlying overexpression of *FGFR3* at the transcriptomic level in CN. Single-copy gene hypomethylation correlating with expression levels has been reported in various types of cancers, and this process has been conjectured to be one of the possible causes of cancer development initiation^73^. *FGFR3* overexpression initiates a series of cascades, including abnormal neuronal precursor cell proliferation, angiogenesis, tumor cell migration, differentiation, survival, and activates pathways such as the PI3K-AKT1 pathway. *FGFR3* pathway activation has also been reported to have potent impacts on cortical and hippocampal lamination, brain size, neuronal differentiation, and axonal pathfinding^74,75^. Additionally, FGFR gene family alterations have been reported frequently in patients with low-grade neuroepithelial tumors^76^. *FGFR3* upregulation has also been observed in CN^11^. Taken together, our findings suggest that CN tumorigenesis is initiated by epigenetic changes rather than by genetic aberrations. Our study paves the way for future functional studies with CN samples to explore and confirm the hypotheses generated through these findings. Interestingly, another well-known type of intraventricular CNS tumor, ependymoma (EPN), with subtypes that lack corresponding genetic mutations, has also been linked to epigenetic changes as a driver of oncogenesis^77,78^. However, unlike CNs, EPNs are heterogeneous tumors that can be divided into various subcategories according to a recent single-cell RNA-seq study^77^. It has been reported that one of the subcategories of EPN driven by *C11orf95-RELA* or *C11orf95-YAP* fusions is enriched in radial glial cells with aberrant neurodevelopmental pathways and characterized by *FGFR3* overexpression followed by epigenetic changes^77^. Although we did not find evidence of any such fusions in CN, the similarities in epigenetically influenced *FGFR3* overexpression in EPN and CN are intriguing and might lead to elucidation of a new mechanism of brain tumor development in future research. Promising results in controlling EPN tumor cell proliferation with FGFR-targeted therapies have been obtained in vitro^77^. This corroborative evidence from research on EPN indicates the potential of anti-*FGFR3* therapy in CN.

**Fig. 5:**
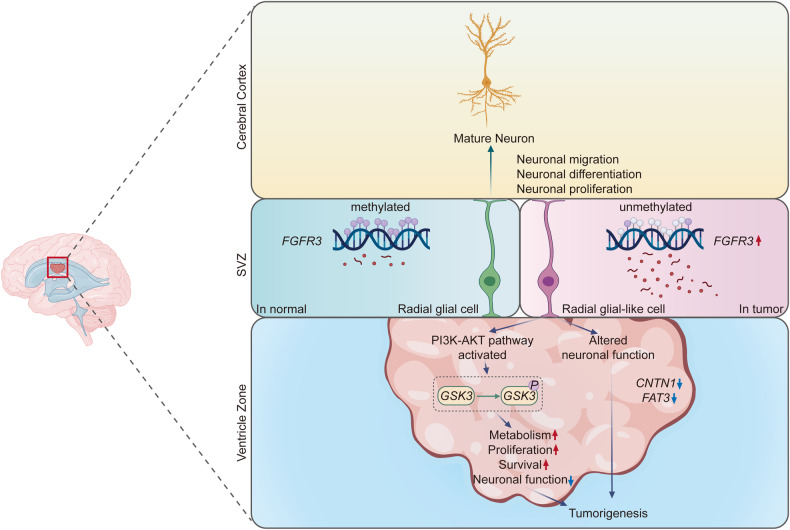
Schematic representation of the potential mechanism of CN tumorigenesis. The middle left and upper panels show the normal course of radial glial cell differentiation into mature neurons. The middle right and lower panels show the potential course of CN tumorigenesis, whereby *FGFR3* hypomethylation-driven *FGFR3*-overexpressing radial glial-like cells progress through the ventricle as a result of PIK3-AKT activation and altered neuronal functions such as differentiation and migration.

Based on our findings, we believe that CN tumorigenesis originates from aberrant radial glial cells in the SVZ, in which *FGFR3* is overexpressed by hypomethylation of CpG sites in the gene. Overexpression of *FGFR3* in these cells might perturb abnormal neural development pathways and activate the oncogenic PI3K-AKT pathway, in addition to downregulating several essential pathways involved in normal neuronal differentiation and migration (Fig. 5). Culmination of all these events leads radial glial-like cells to deviate from their natural course of differentiation and migration in which they become mature neurons and instead end up in the ventricles, from which they develop into CN. These supporting results reinforce our hypothesis that *FGFR3* plays a crucial role in CN tumorigenesis.

Despite its limited sample size, our study adds a significant amount of new data to enhance the current knowledge of the very rare CNS tumor CN. Moreover, there are multiple possible future research directions, including but not limited to whole-genome studies, to further explore the less likely presence of genomic drivers in CN; transcriptomic and single-cell studies with increased CN sample numbers; and functional validation of *FGFR3* as the key driver in CN. Furthermore, the specific mechanism by which hypomethylation of *FGFR3* drives radial glial cells to deviate from their natural differentiation course to instead give rise to CN inside ventricles will require further exploration via large-scale methylation studies of CN in the future.

In conclusion, our study establishes a working hypothesis for an epigenetic cause of tumorigenesis without causing a gene mutation in CN and identifies the PI3K-AKT pathway as a key oncogenic pathway. Hypomethylation of the *FGFR3* promoter and its overexpression present promising potential as emerging biomarkers and treatment targets in the CN patients.

### Supplementary information

Supplementary Tables

Supplementary Figures

## Data Availability

All sequencing files have been deposited in the NCBI short-read sequence archive (https://www.ncbi.nlm.nih.gov/sra) under BioProject number PRJNA796513. The processed data, including gene mutation, expression, and fusion information, are provided in the Supplementary Tables of the manuscript.
