# Supplementary material for: Central neurocytoma exhibits radial glial cell signatures with FGFR3 hypomethylation and overexpression
Source: Exp Mol Med. 2024 Apr 12;56(4):975–86. doi: 10.1038/s12276-024-01204-3 (PMC11059271; doi:10.1038/s12276-024-01204-3)
Supplement: Supplementary file 2 — Supplementary Figures [file 12276_2024_1204_MOESM2_ESM.pdf]

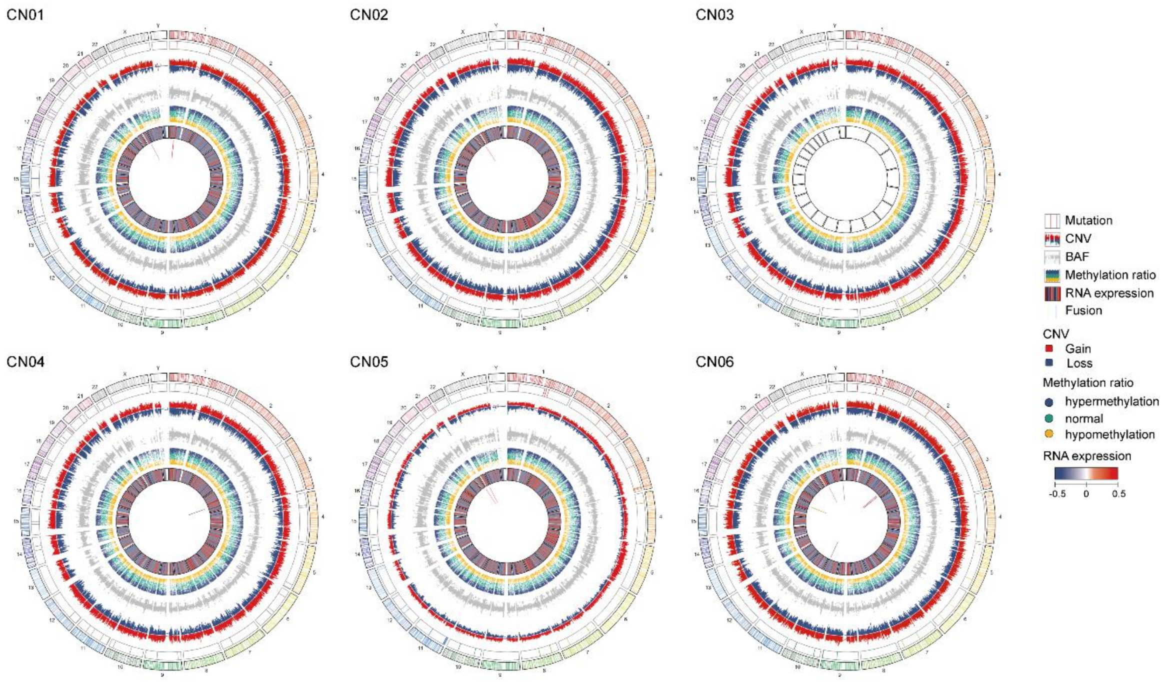

Supplementary Fig. 1 Circos plot of 6 CN tumor sample genetic profiles showing absence of any repeated SNPs, CNVs or gene fusions. From outwards to inwards the circles represent the chromosome guide, SNP mutation, CNV profile, B allele frequency (BAF), methylation ratio, RNA expression and gene fusion information of each sample

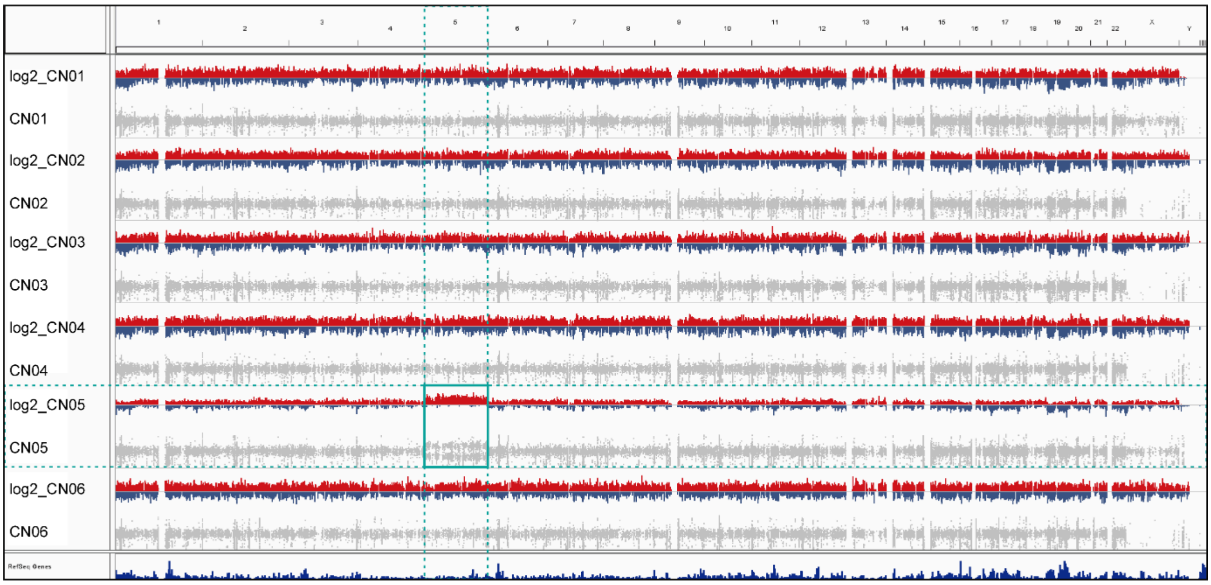

Supplementary Fig. 2 CNV profile of CN cases. CNV profiles of CN samples showing the absence of repeated gains or losses or LOH across 6 samples

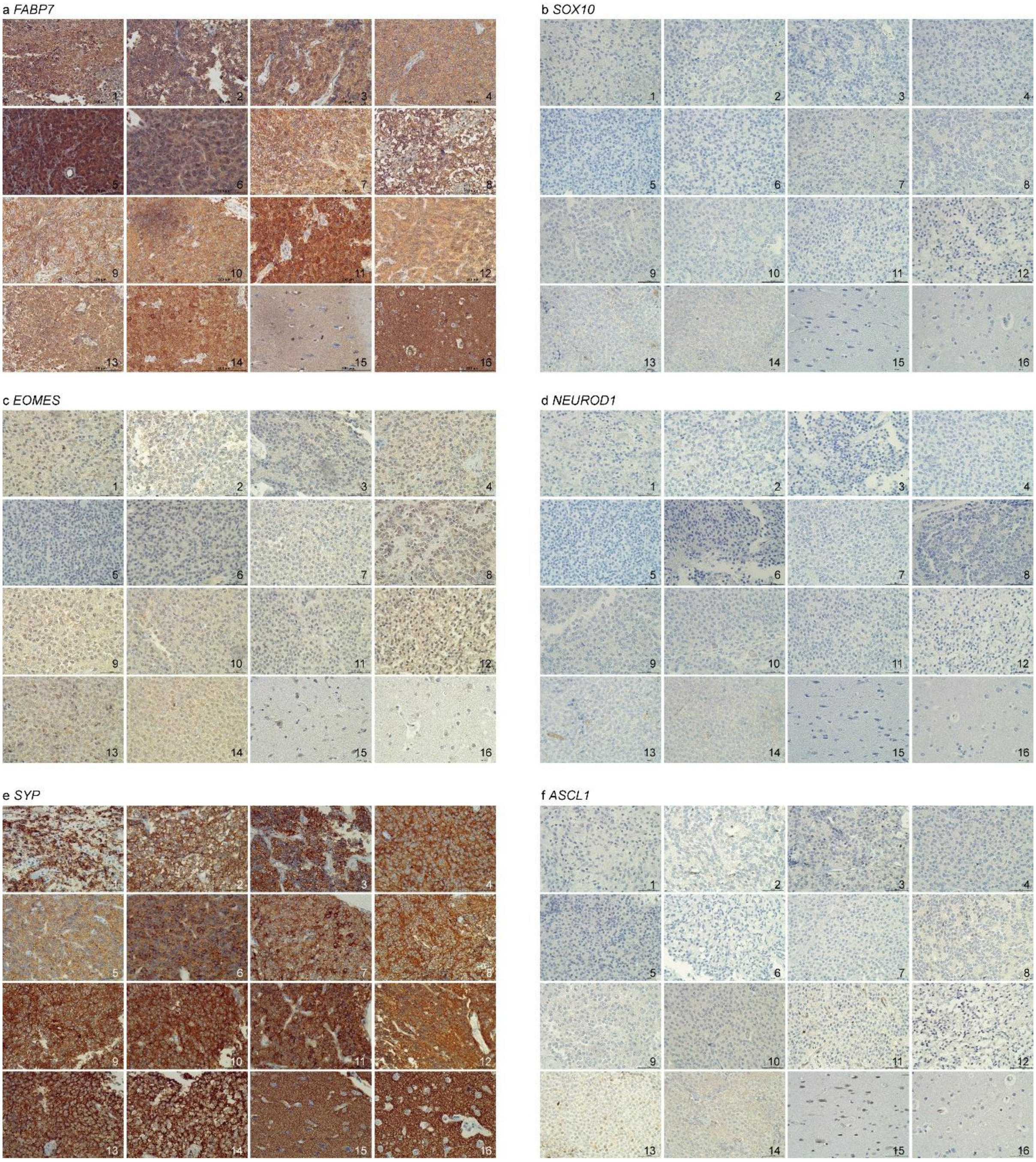

Supplementary Fig. 3 IHC validation with the CN tissue array. IHC of the *FGFR3, PIK3R3, AKT1, GSK3, SOX2, PAX6, FABP7, SOX10, EOMES, NEUROD1, SYP, and ASCL1* genes in 14 CN (1-14) and 2 normal brain (15-16) tissue array slides (Scale bar 100 uM)

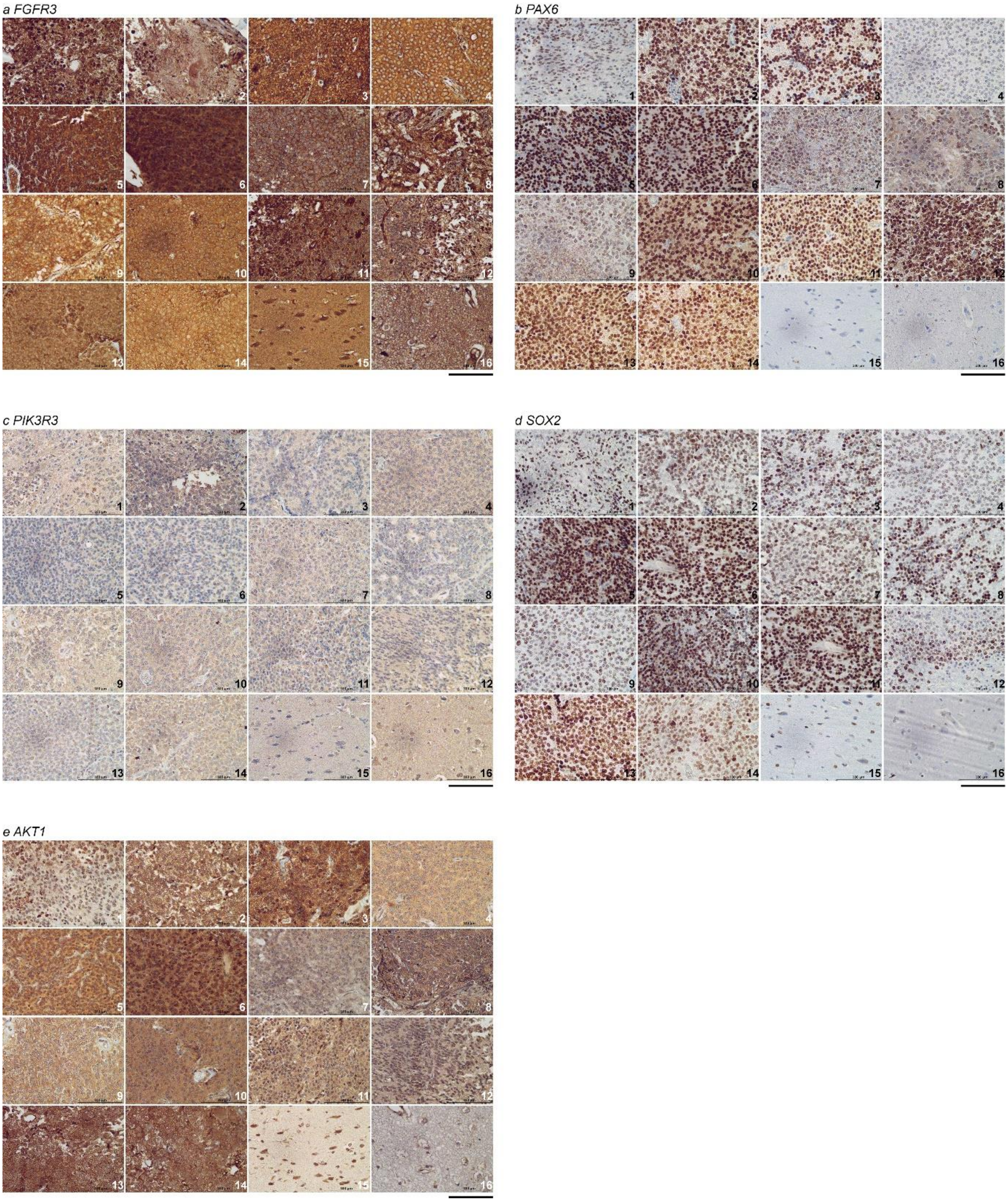

Supplementary Fig. 3 continued.

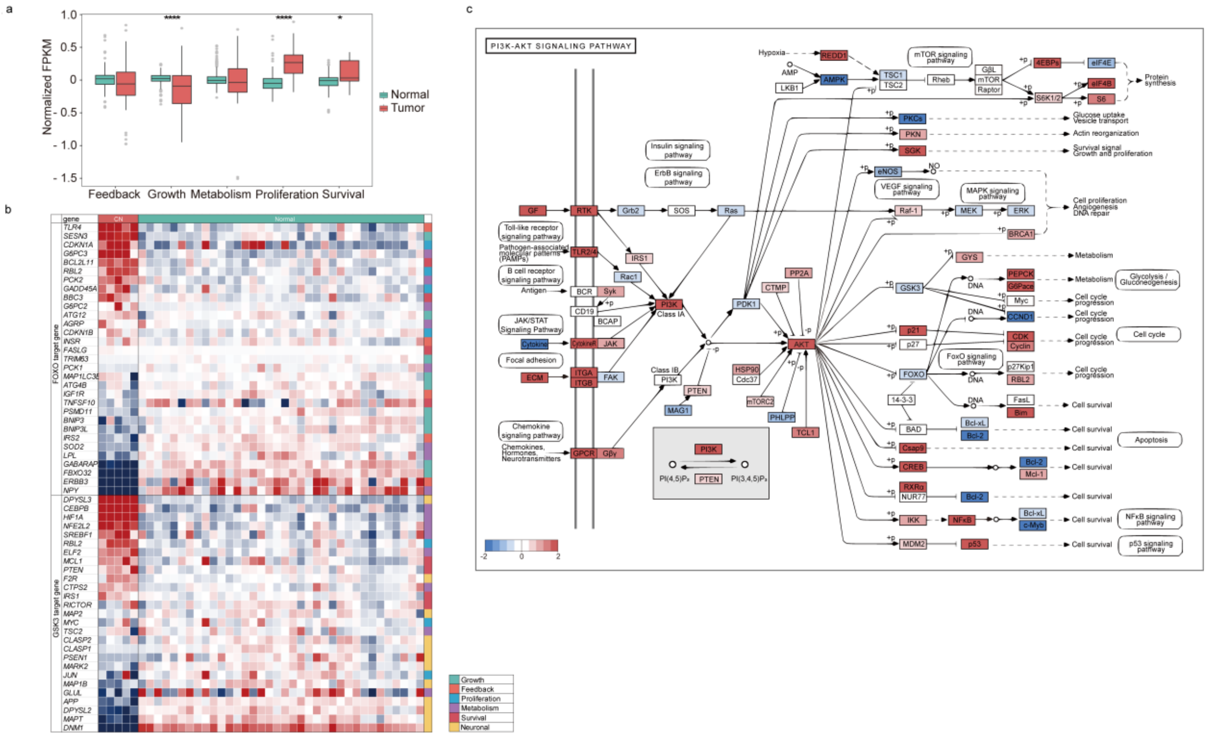

Supplementary Fig. 4 Confirmation of PI3K-AKT pathway downstream activation a. Comparison of expression between CN and normal brain tissues in functional groups related to *FOXO* b. Heatmap showing genes related to the downstream activation of the PI3K-AKT pathway (*FOXO* and *GSK3*-related genes) in CN and normal brain samples c. Schematic representation of the PI3K-AKT pathway showing downstream pathways and genes. Upregulated and downregulated genes are color-coded in red and blue respectively

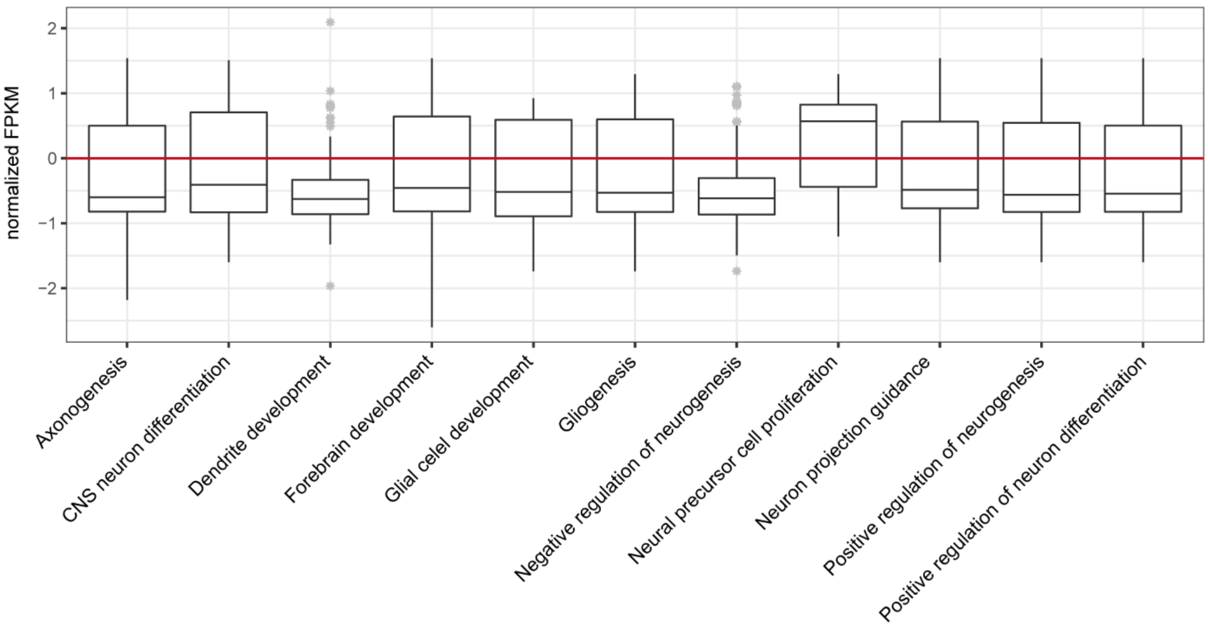

Supplementary Fig. 5 Downregulation of neurodevelopmental pathways in CN. Expression of gene sets related to neurodevelopmental pathways showing the downregulation of neuron differentiation, neurogenesis, neuron projection guidance and upregulation of neural precursor cell proliferation in CN

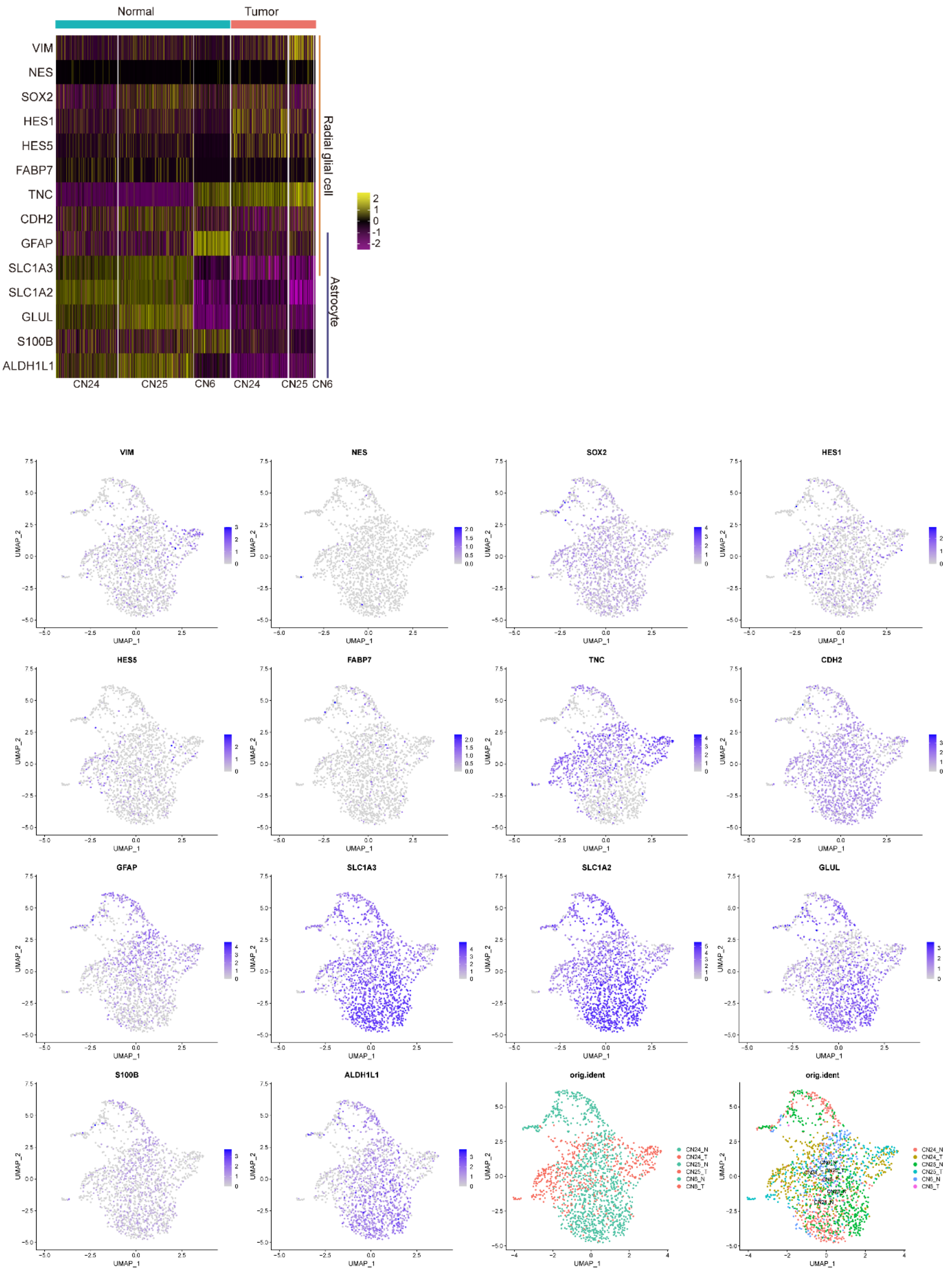

Supplementary Fig. 6 Radial glial/astrocyte-like cell cluster expression pattern a. heatmap of astrocyte and radial glial cell marker b. Feature plot of isolated cluster 5 with astrocyte and radial glial cell marker

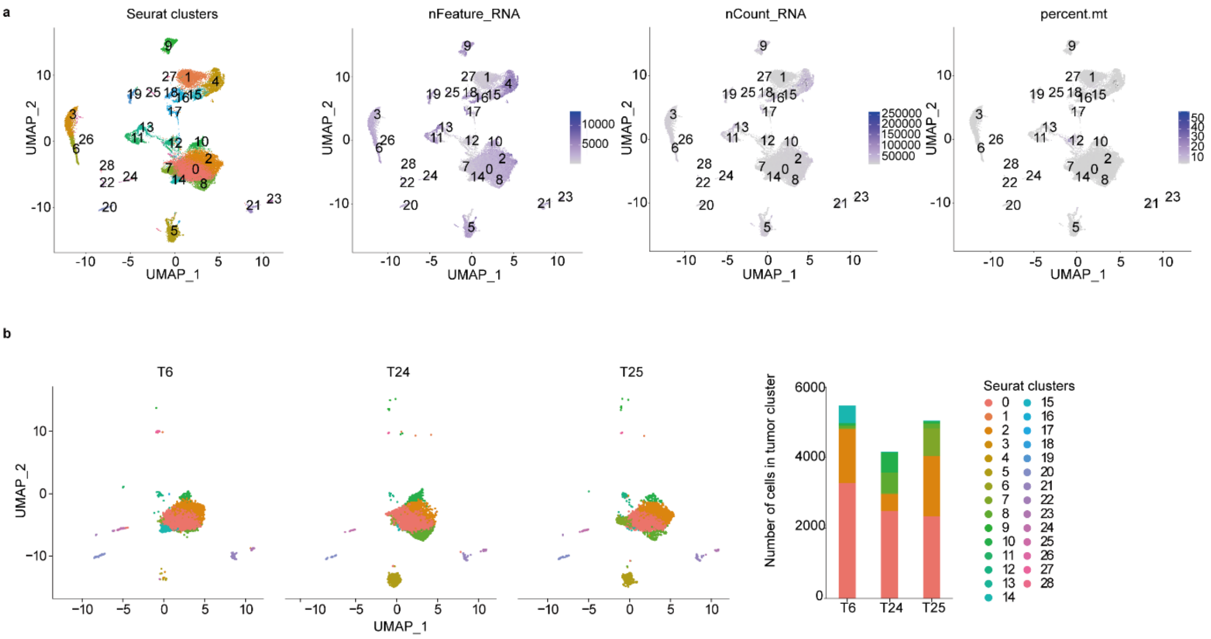

Supplementary Fig. 7 CN tumor cells are homogenous a. Seurat clusters of all cells based on snRNA-seq showing gene numbers, counts and mitochondrial DNA percentages in each cluster b. Tumor cell specific clusters (0, 2, 7, 8, 10, and 14) in 3 CN samples showing that major clusters (0, 2, and 10) are common between the samples. Sample specific clusters (7, 8, and 14) are minimal and more likely to represent sample biased cells

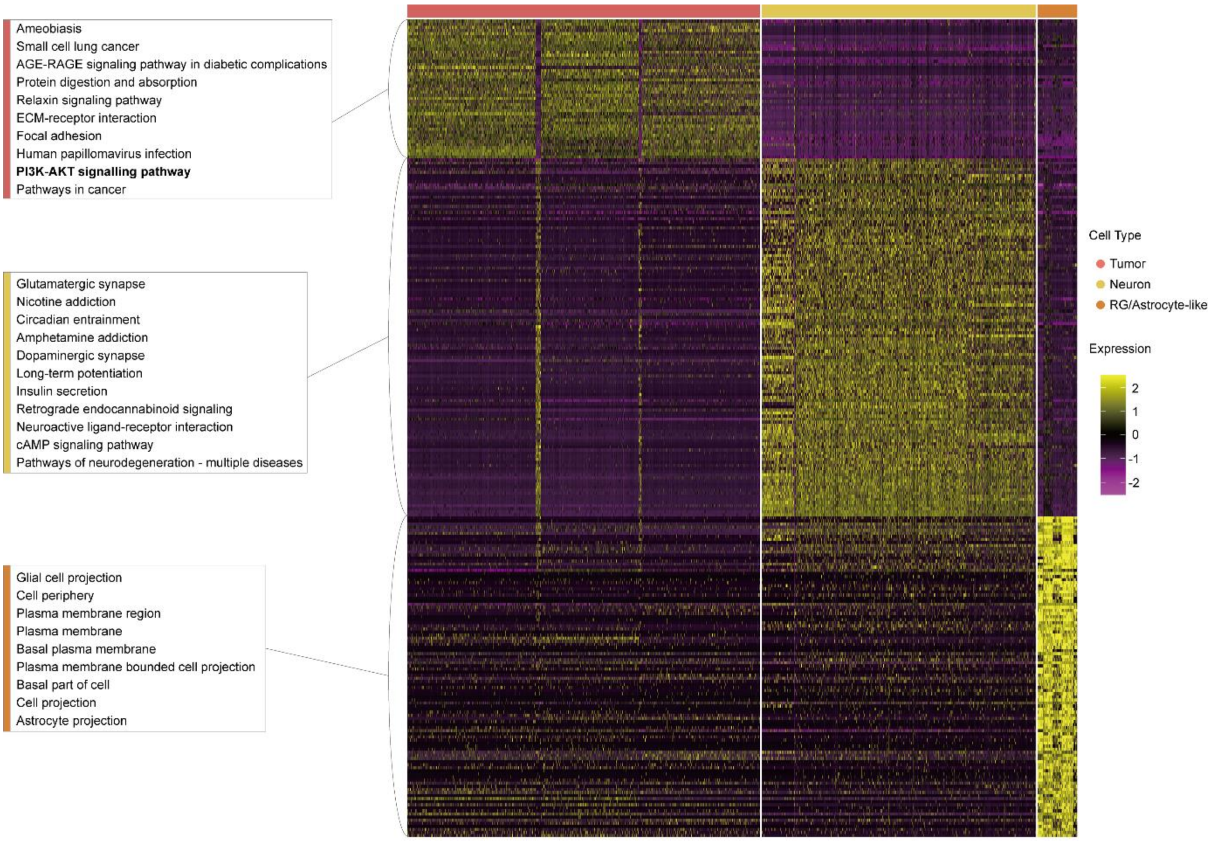

Supplementary Fig. 8 Heatmap of tumor, neuron and radial glial/astrocyte-like enriched genes showing associations with the PI3K-AKT signaling pathway

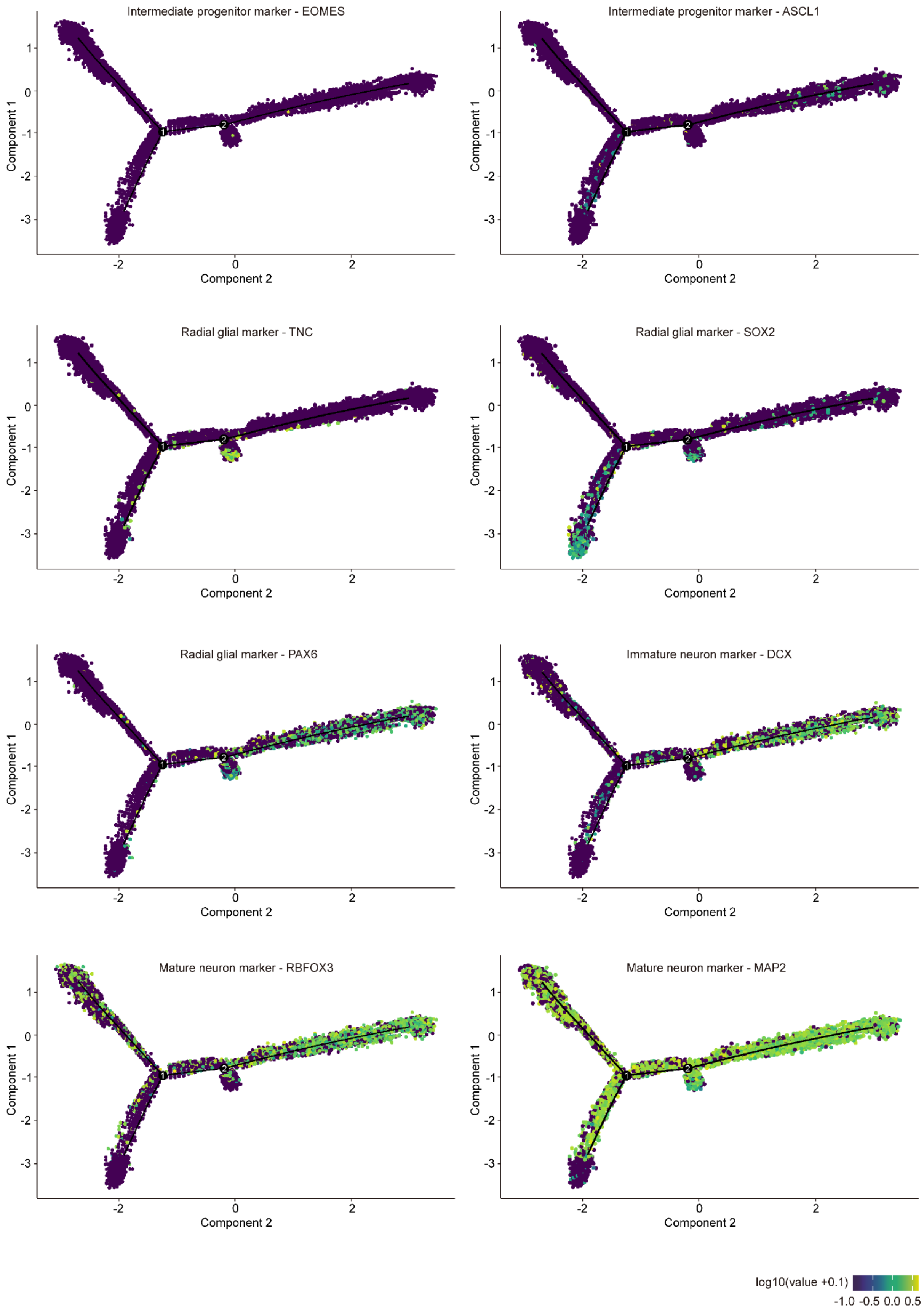

Supplementary Fig. 9 Identifying the cell type of tumor cluster with radial glial, intermediate progenitor, immature neuron, and mature neuron cells

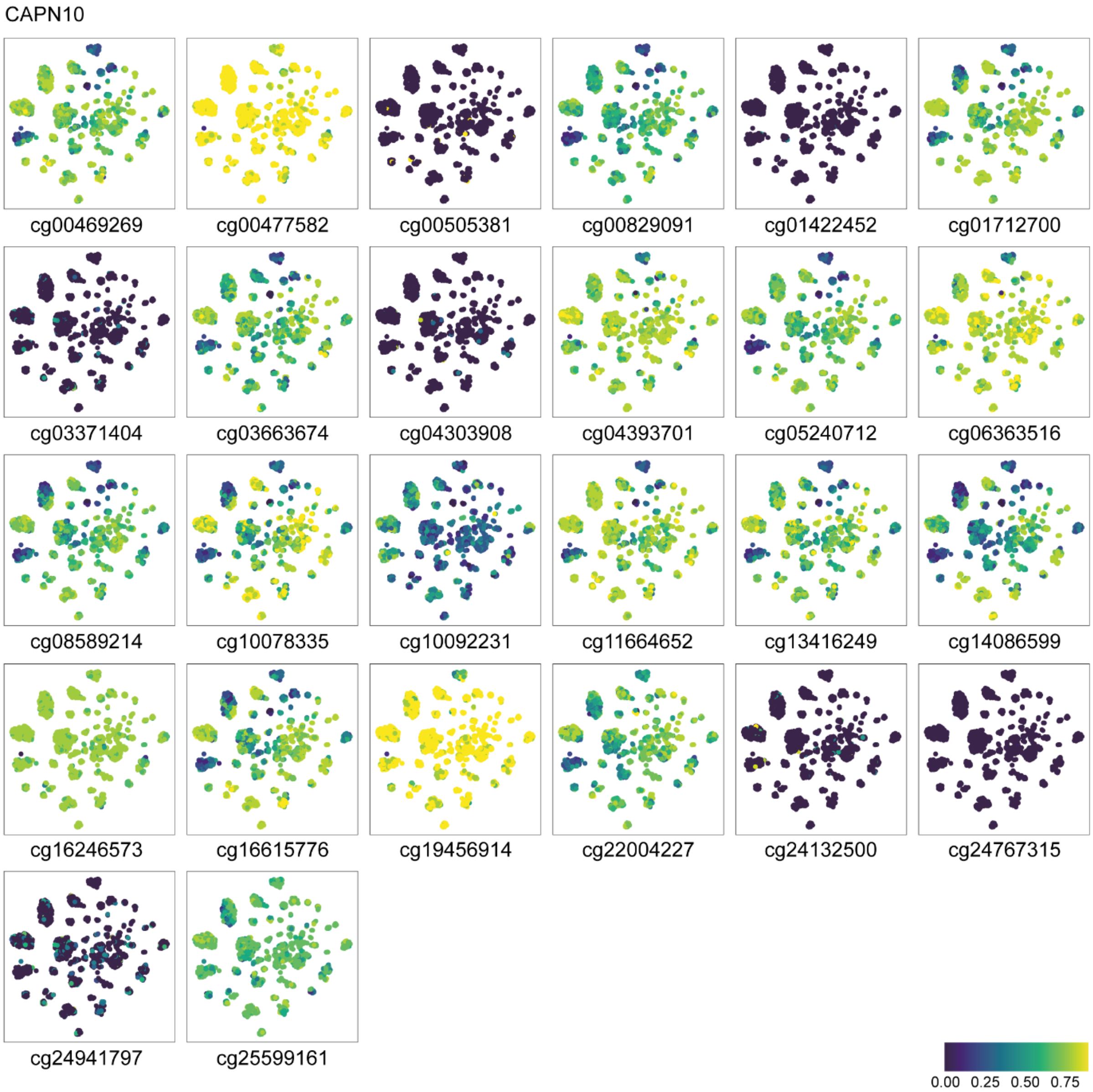

Supplementary Fig. 10 *CAPN10* gene CpG island methylation expression in CNS tumors, including CN

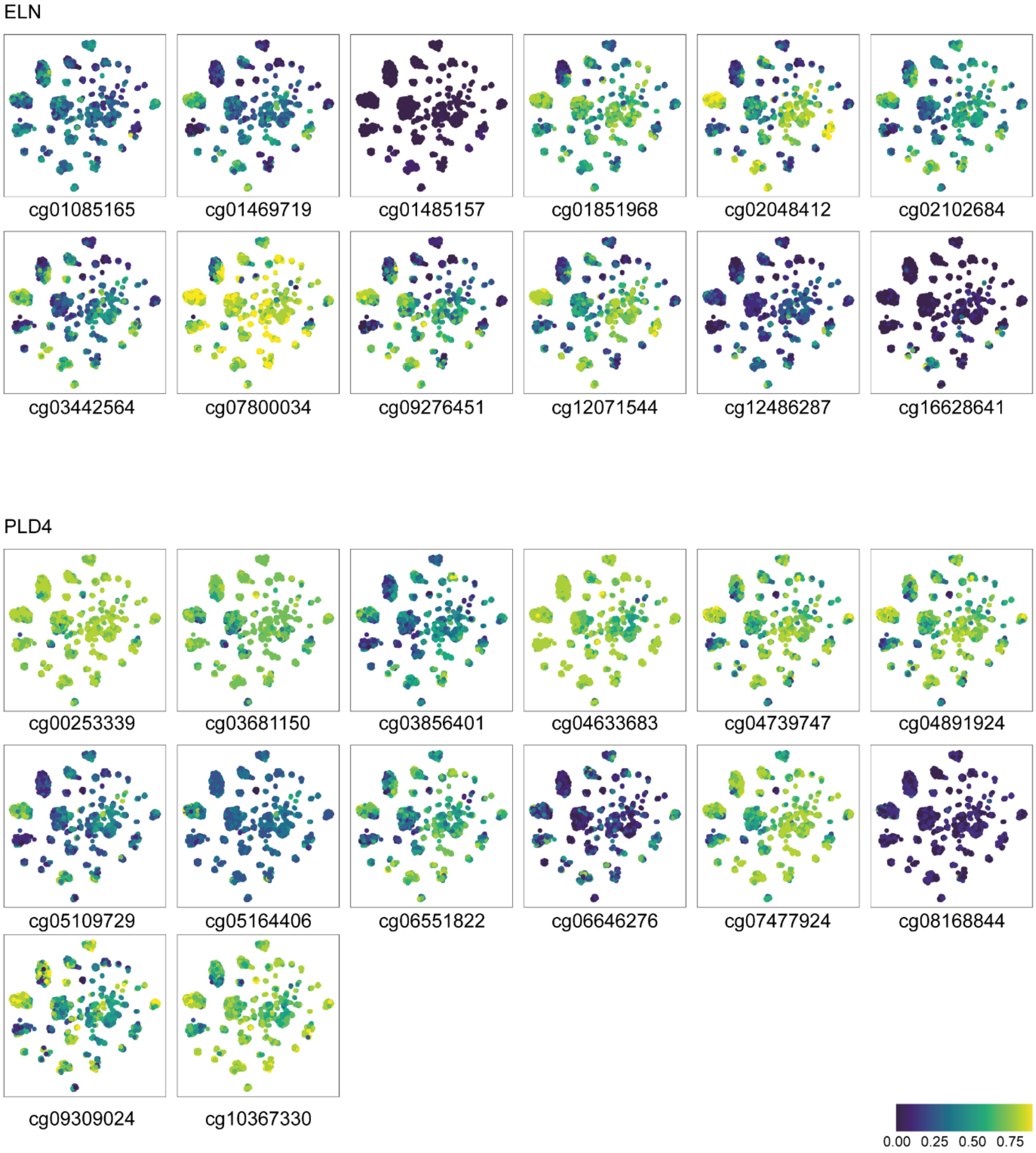

Supplementary Fig. 10 *ELN* and *PLD4* gene CpG island methylation expression in CNS tumors, including CN

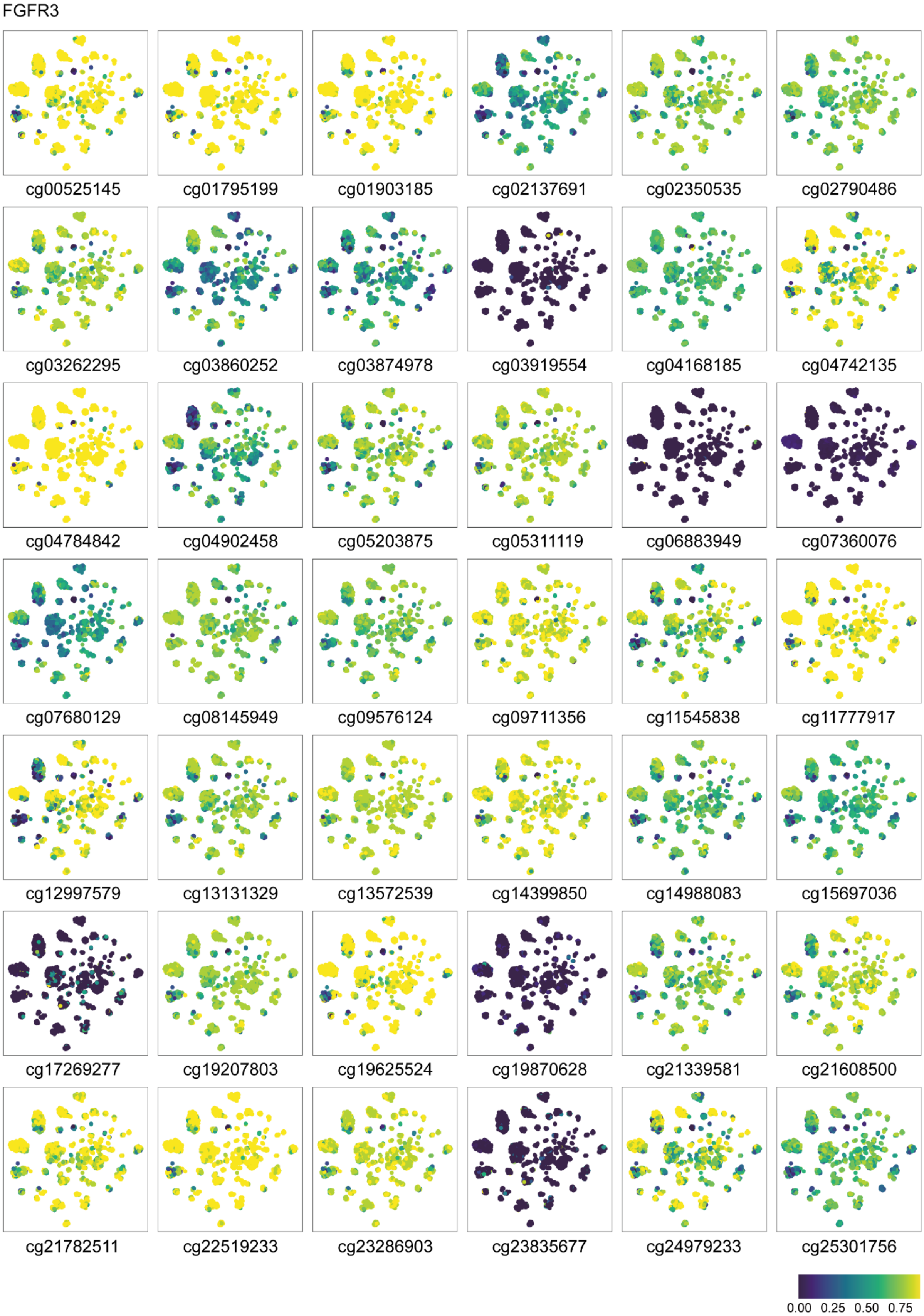

Supplementary Fig. 10 *FGFR3* gene CpG island methylation expression in CNS tumors, including CN

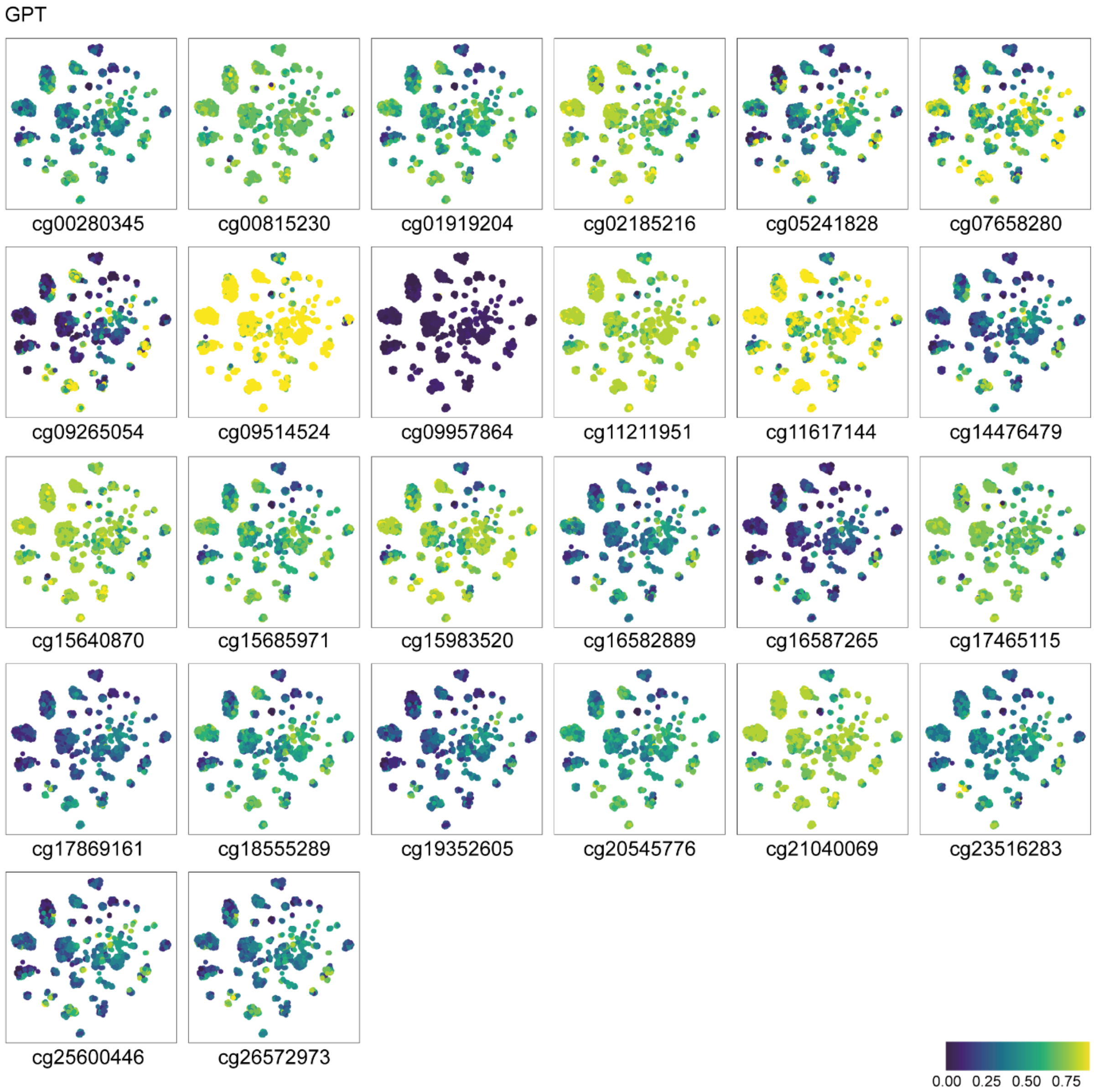

Supplementary Fig. 10 *GPT* gene CpG island methylation expression in CNS tumors, including CN

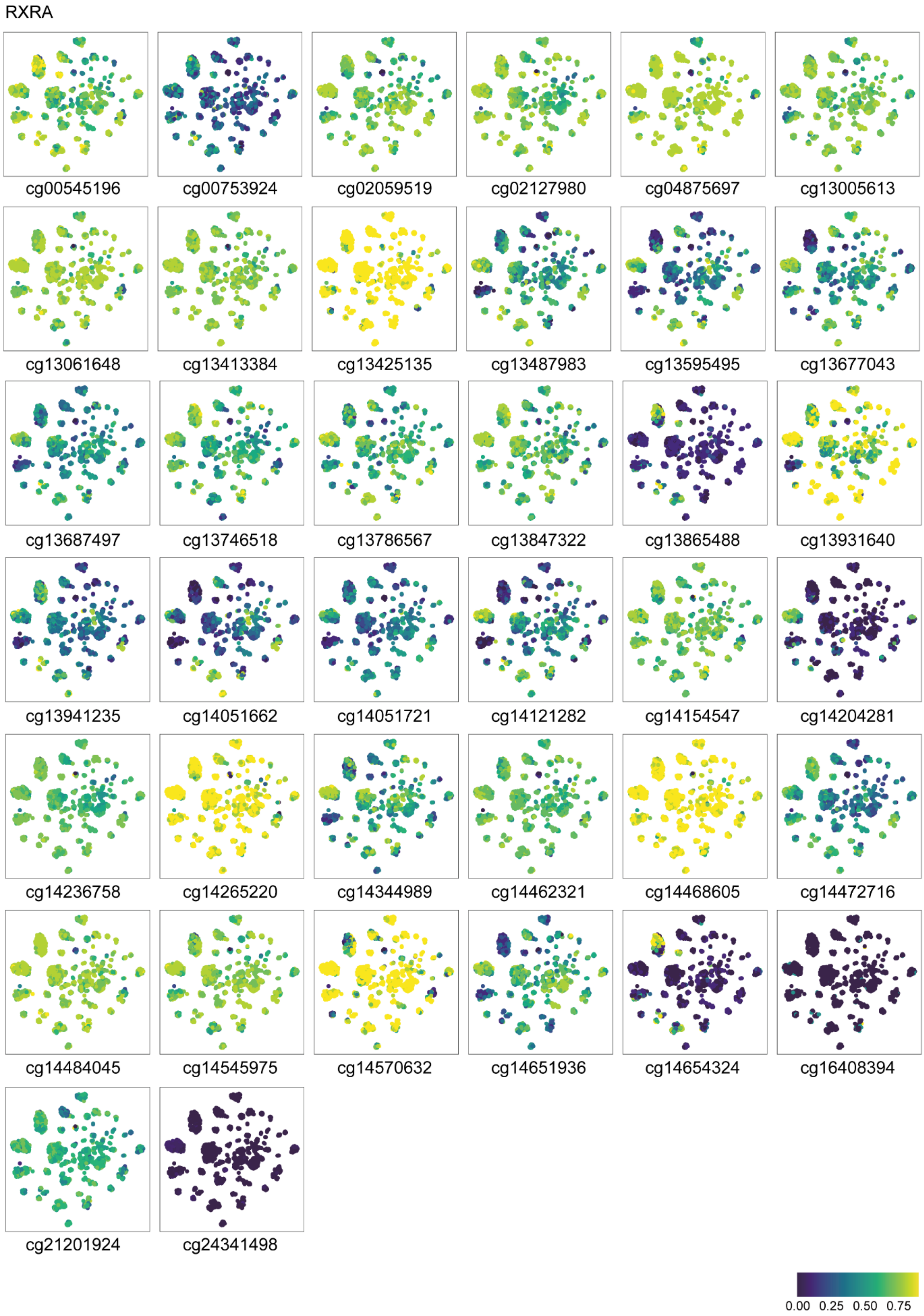

Supplementary Fig. 10 *RXRA* gene CpG island methylation expression in CNS tumors, including CN

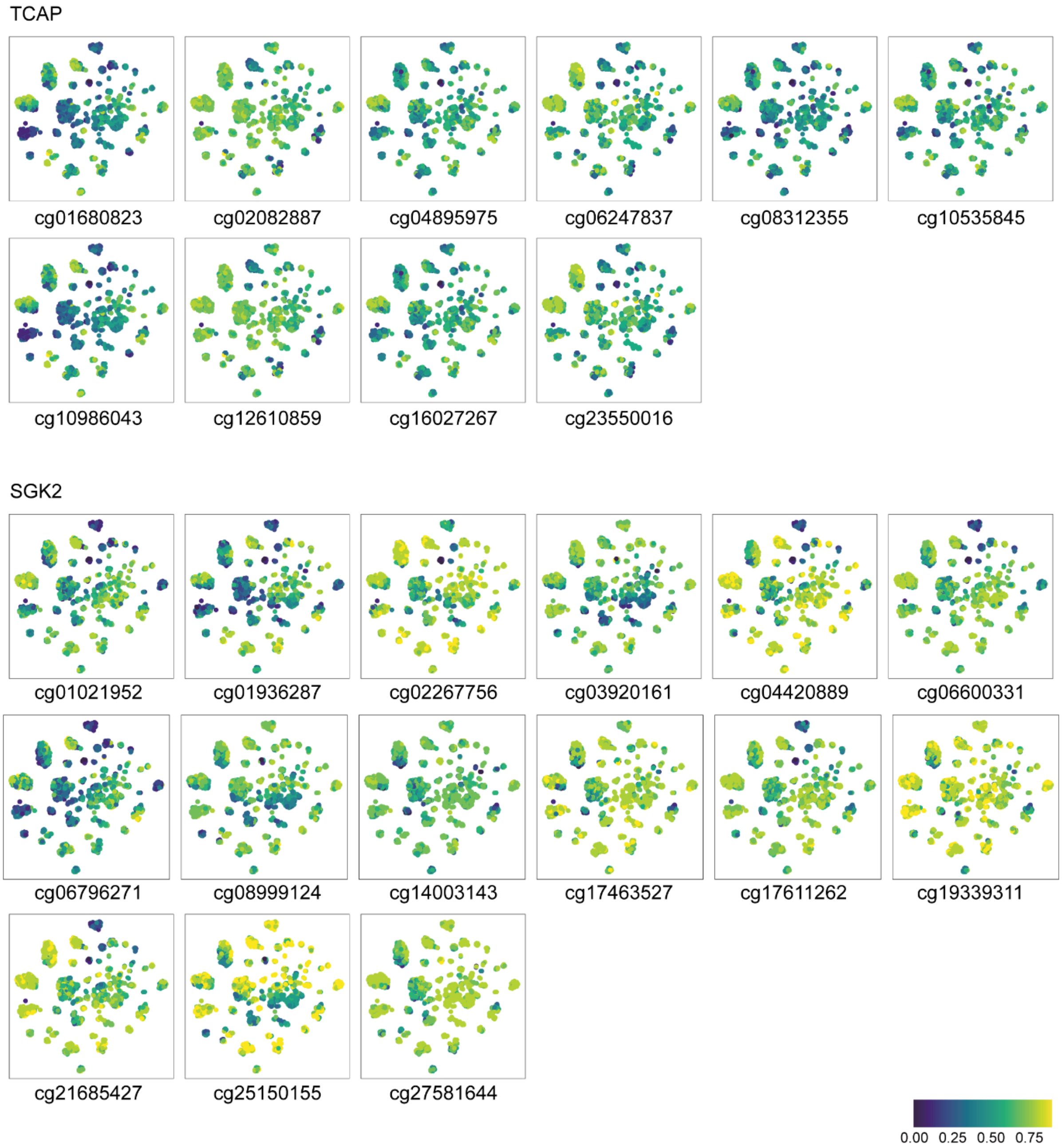

Supplementary Fig. 10 *TCAP* and *SGK2* gene CpG island methylation expression in CNS tumors, including CN

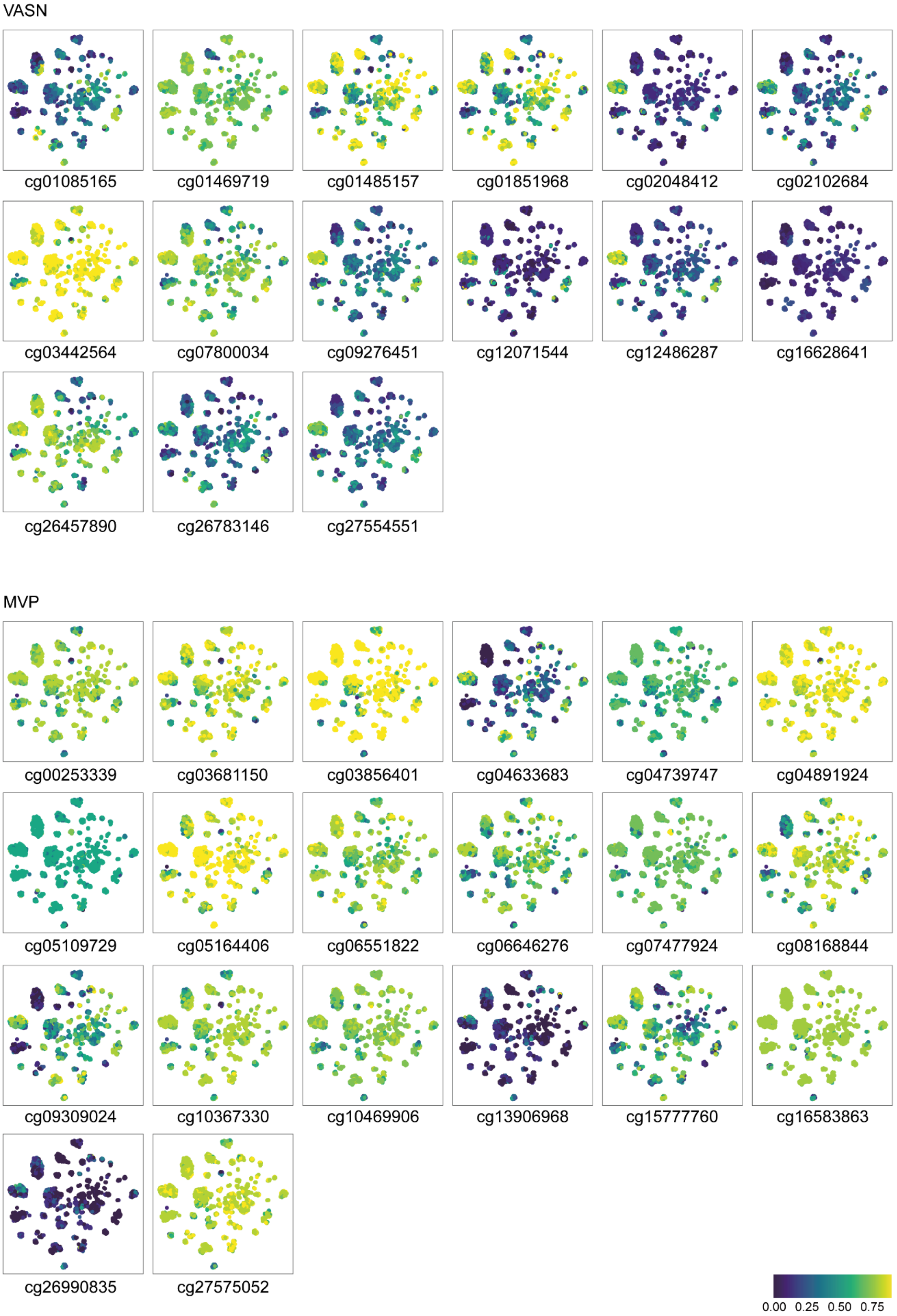

Supplementary Fig. 10 *VASN* and *MVP* gene CpG island methylation expression in CNS tumors, including CN

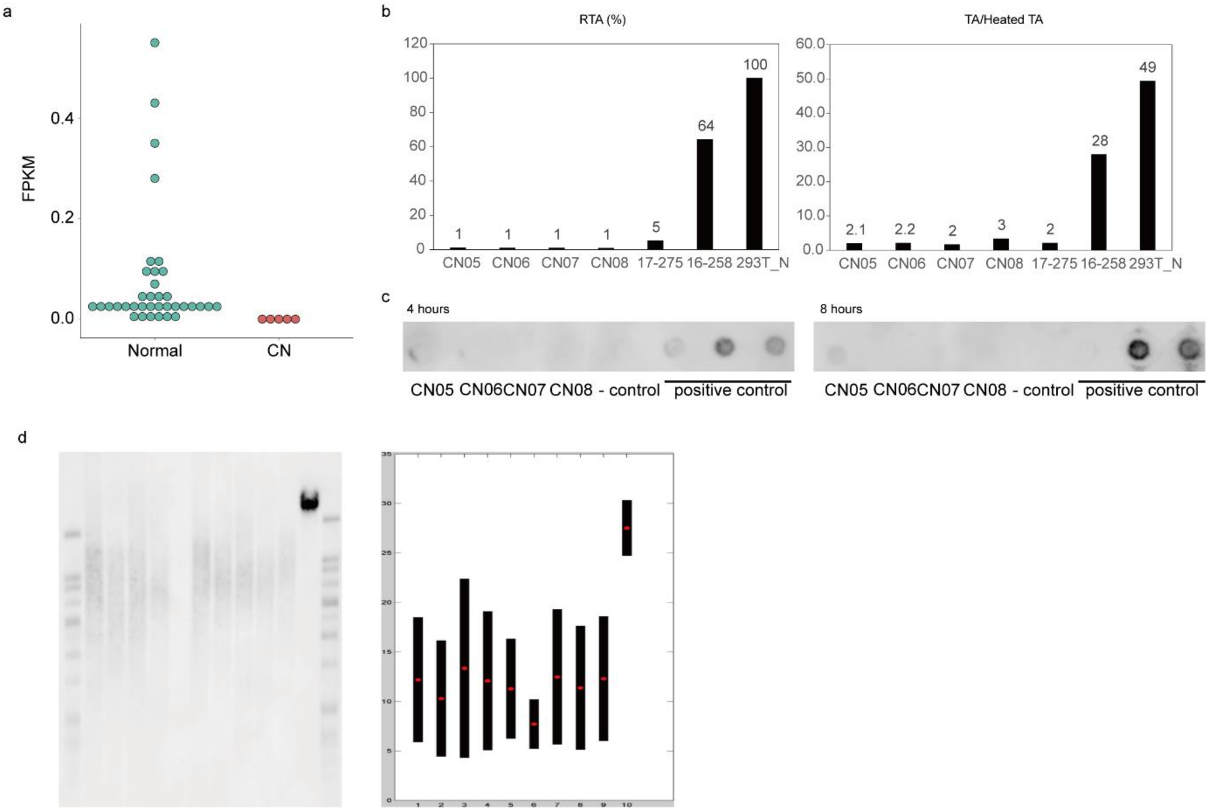

Supplementary Fig. 11 Telomere maintenance mechanism in CN a. *TERT* gene expression was absent in all the CN samples compared to normal brain samples b. TRAP assay showing minimal telomerase activity in CN compared to the positive control c. C-circle assay showing negative result for alternate telomere activity (ALT) d. TRF assay showing a mean telomere length between 9-13 kb in CN
